# Supplementary material for: Genome-wide CRISPR screens identify the YAP/TEAD axis as a driver of persister cells in EGFR mutant lung cancer
Source: Commun Biol. 2024 Apr 24;7:497. doi: 10.1038/s42003-024-06190-w (PMC11043391; doi:10.1038/s42003-024-06190-w)
Supplement: Supplementary file 2 — Supplementary Information [file 42003_2024_6190_MOESM2_ESM.pdf]

Supplementary Figure 1

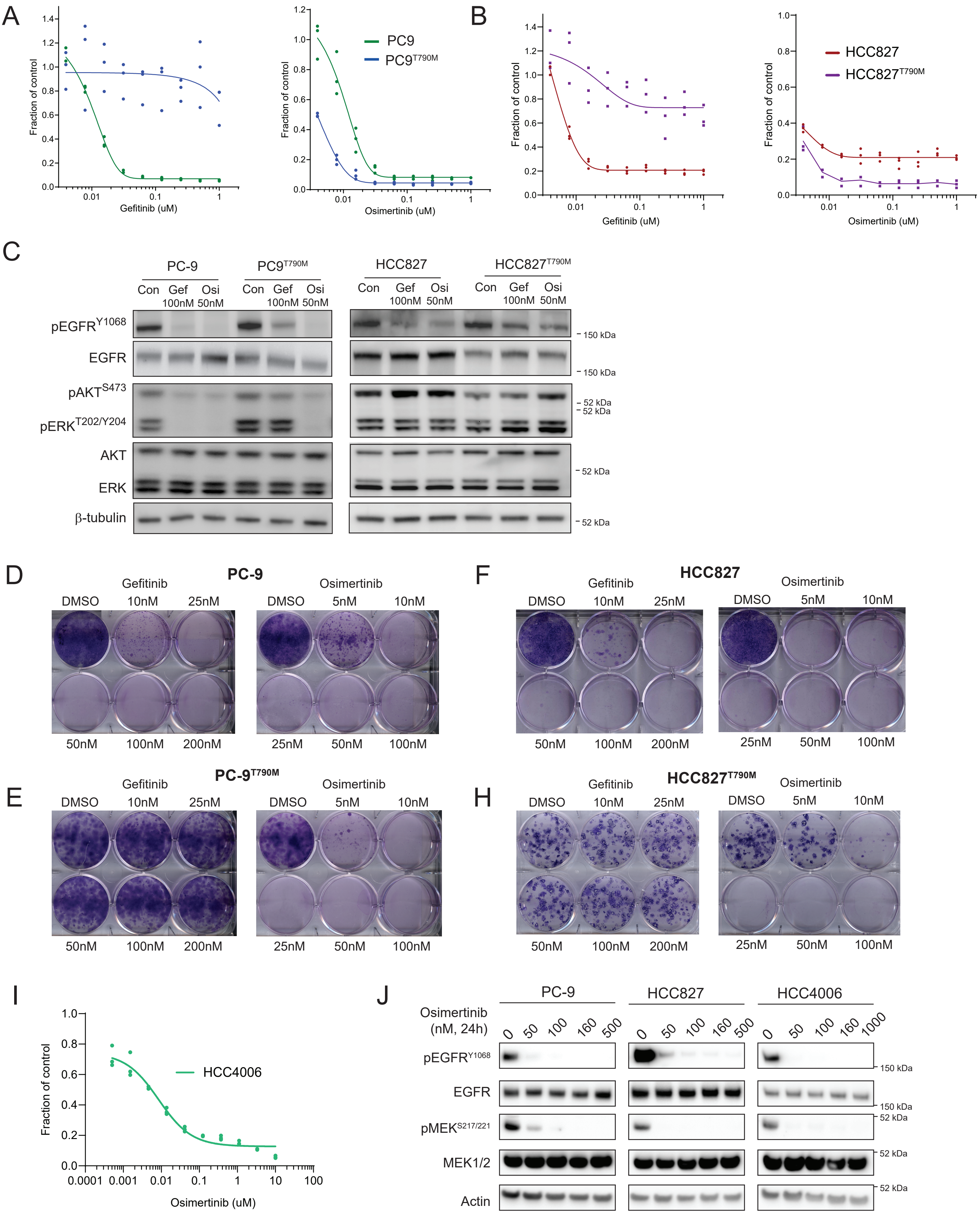

Supplementary Figure 1. (A,B) Dose response curves of gefitinib and osimertinib in PC-9, PC-9^{T790M}, HCC827 and HCC827^{T790M} cell lines. Viability was measured at day 6. (C) Immunoblots of effect of treating cell lines with gefitinib (100nM) or osimertinib (50nM) for 2 hours. (D-H) Clonogenic survival assays in cell lines treated with a dose range of gefitinib or osimertinib over 21 days. (I) Dose response curve of osimertinib in HCC4006 cells. Viability was measured at day 6. (J) Immunoblots of effect of treating cell lines with a concentration range of osimertinib at 24 hours.

Supplementary Figure 2

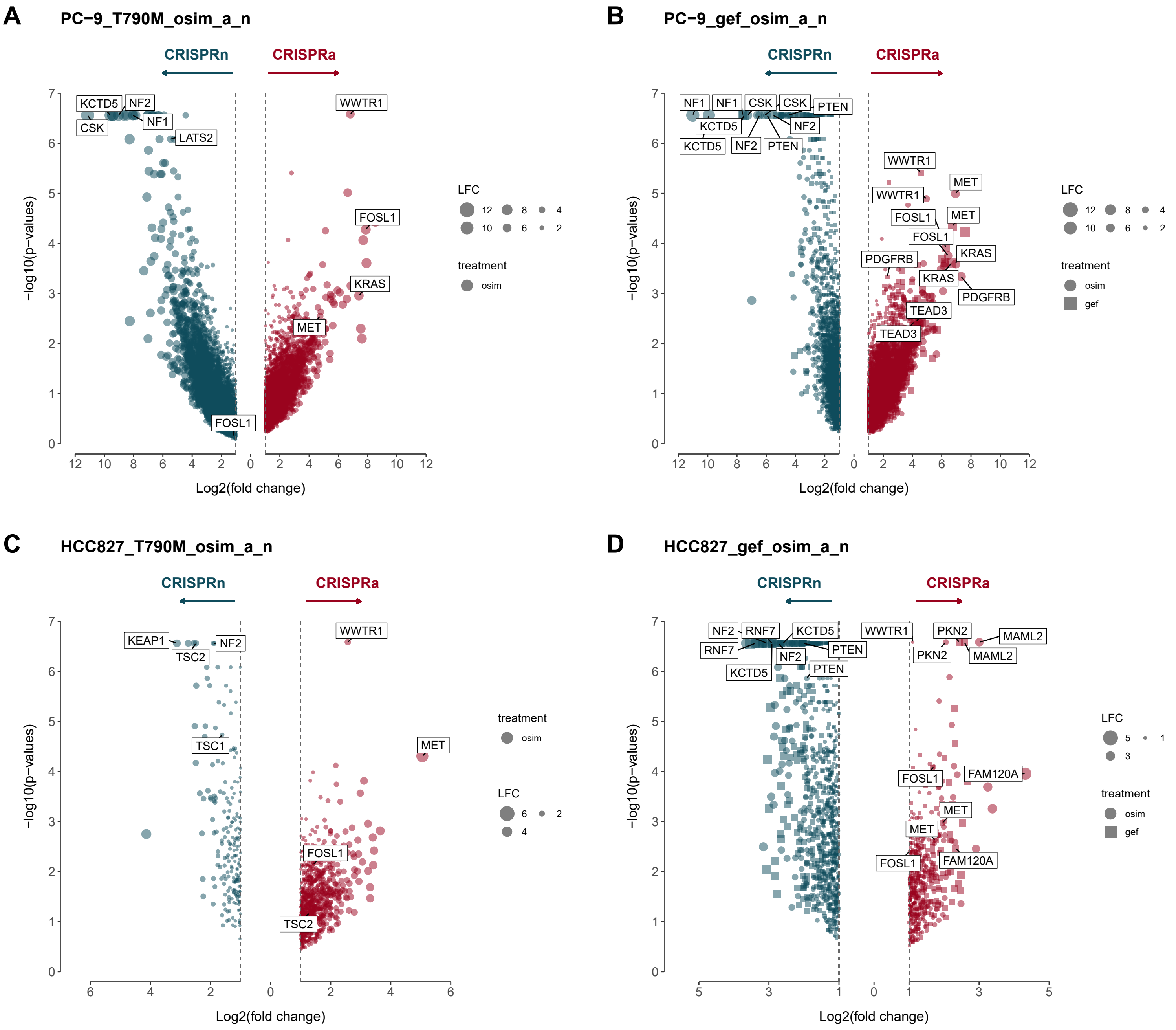

Supplementary Figure 2. Volcano plots of resistance genes identified from genome-wide CRISPR knockout (CRISPRn, blue shapes) or activation (CRISPRa, red shapes) screens in EGFR mutant lung cancer cell lines. Only those genes with >= absolute 2-fold resistance effect are shown. Genes of particular signifiance or highly recurrent are indicated. The size of each circle/square is proportional to the Log Fold Change (LFC) resistance effect size.

Supplementary Figure 3

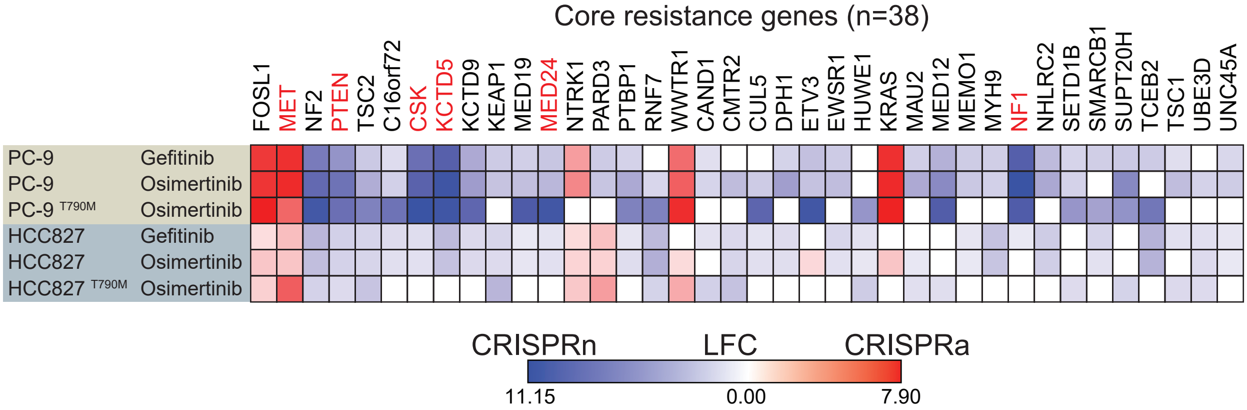

Supplementary Figure 3. Heatmap of recurrent ‘core’ resistance genes (detected in >=4 of 6 experiments). Genes highlighted in red were experimentally validated in PC-9, HCC827 and HCC4006 *EGFR* mutant lung cancer cell lines.

Supplementary Figure 4

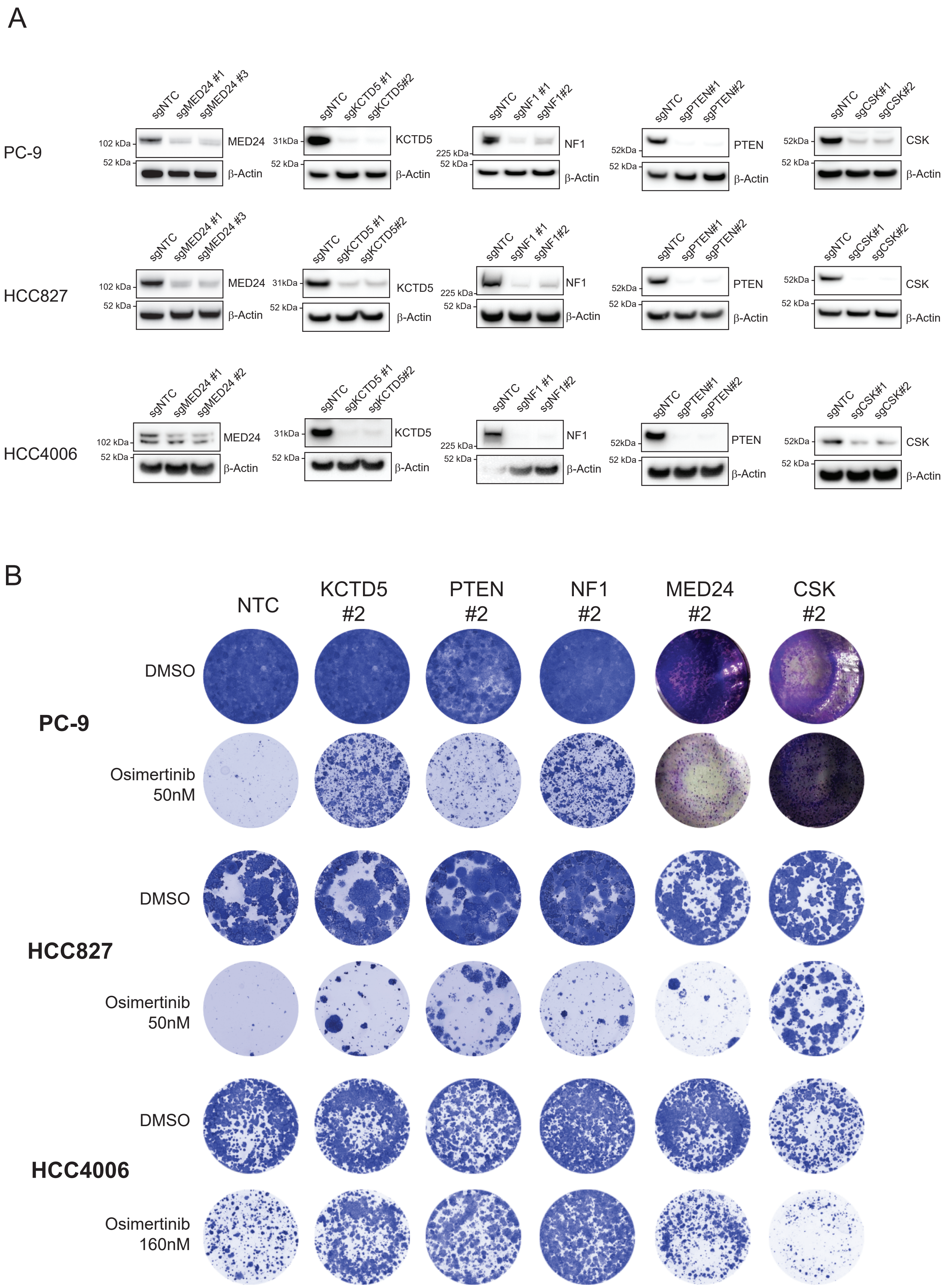

Supplementary Figure 4. (A) Western blots to confirm gene silencing in PC-9, HCC827 and HCC4006 cell lines. NTC = non-targeting control gRNA. (B) Clonogenic survival assays in cell lines (NTC vs gene KO) treated for 21 days with osimertinib.

Supplementary Figure 4 (contd)

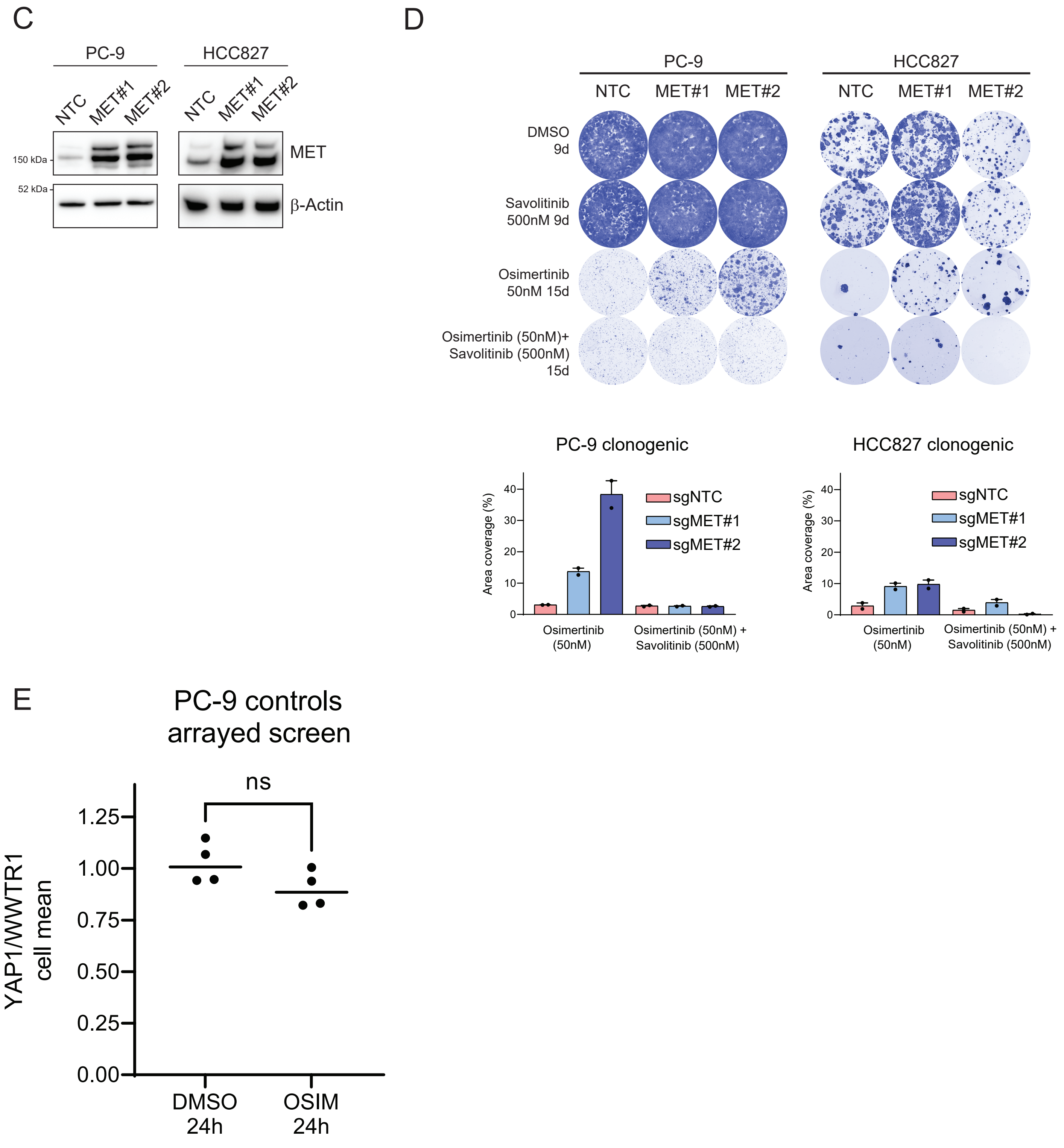

Supplementary Figure 4. (C) Western blot to confirm MET overexpression in PC-9 and HCC827 cells using the CRISPRa SAM system. (D) Clonogenic survival assays of resistance effect of MET overexpression and combination effect of osimertinib and the MET inhibitor savolitinib on viability. (E) Total YAP1/WWTR1 IF expression in PC-9 cells before and after treatment with osimertinib (50nM).

Supplementary Figure 5

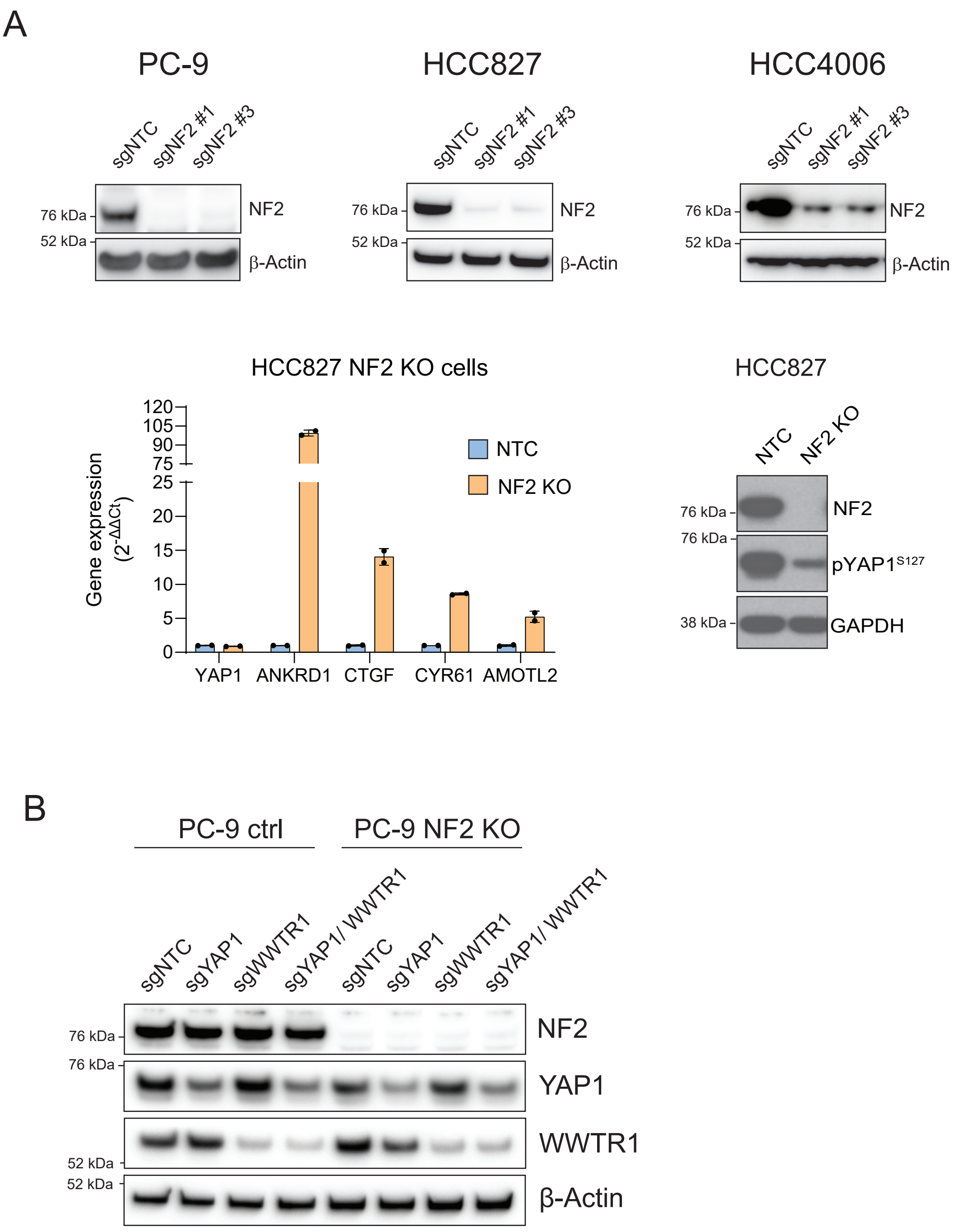

Supplementary Figure 5. (A) (upper panels) Western blots confirming effect on protein expression of silencing NF2 by gRNA in *EGFR* mutant lung cancer cell lines. (A) (lower panels) Effect of NF2 KO in HCC827 cells on mRNA expression of Hippo target genes as well as on levels of phospho-YAP1 (S127). (B) Western blot of NF2, YAP1 and WWTR1 expression following CRISPR KO of YAP1, WWTR1 or both genes together in PC-9 or PC-9/NF2 ko cell lines.

Supplementary Figure 6

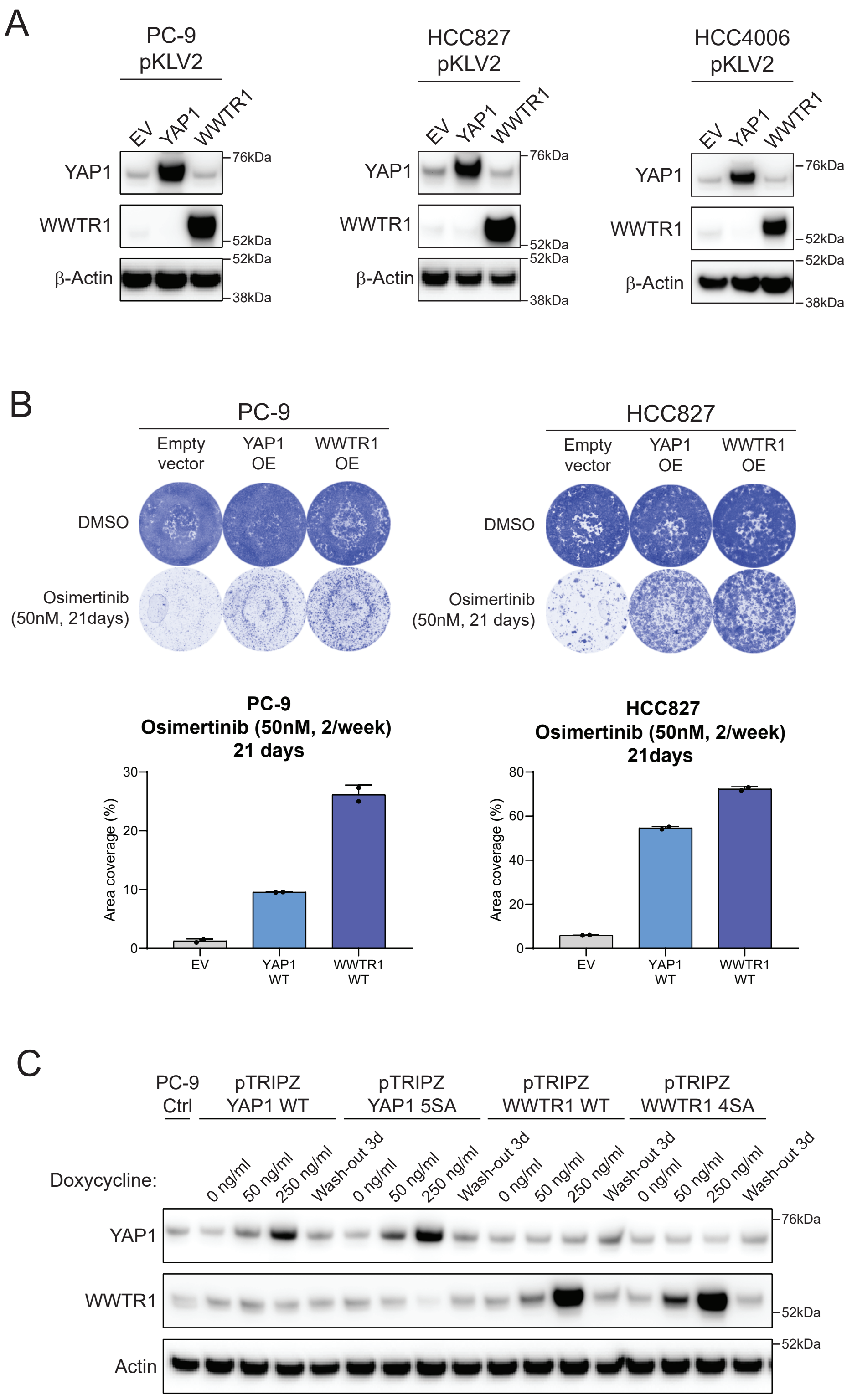

Supplementary Figure 6. (A) Western blots conforming effect on protein expression of over-expressing YAP1 or WWTR1 in *EGFR* mutant lung cancer cell lines. (B) Clonogenic survival assays of YAP1 and WWTR1 overexpressing PC-9 and HCC827 cell lines following treatment for 21 days with osimertinib. (C) Dose-dependent effect of doxycycline on protein expression of wild-type YAP1, wild-type WWTR1, YAP1 5SA mutant or WWTR1 4SA mutant (following treatment with 0, 50 or 250ng/ml doxycycline for 3 days) in PC-9 cells. The effects on expression following a wash out period of 3 days is also shown.

Supplementary Figure 7

A

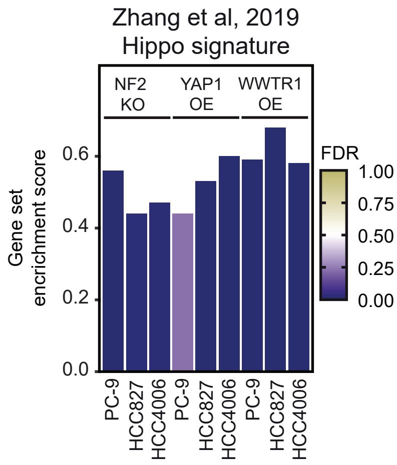

B

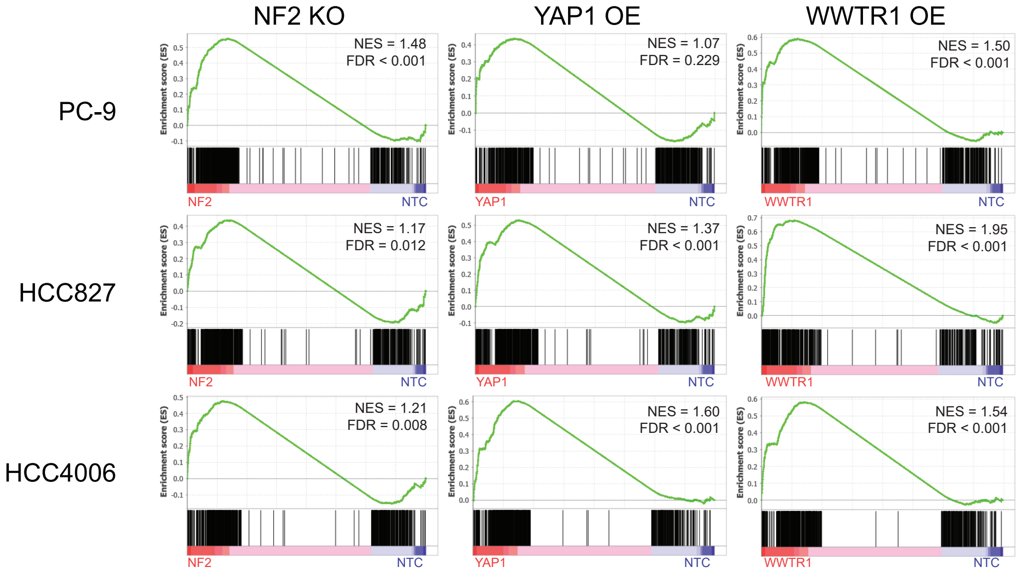

C

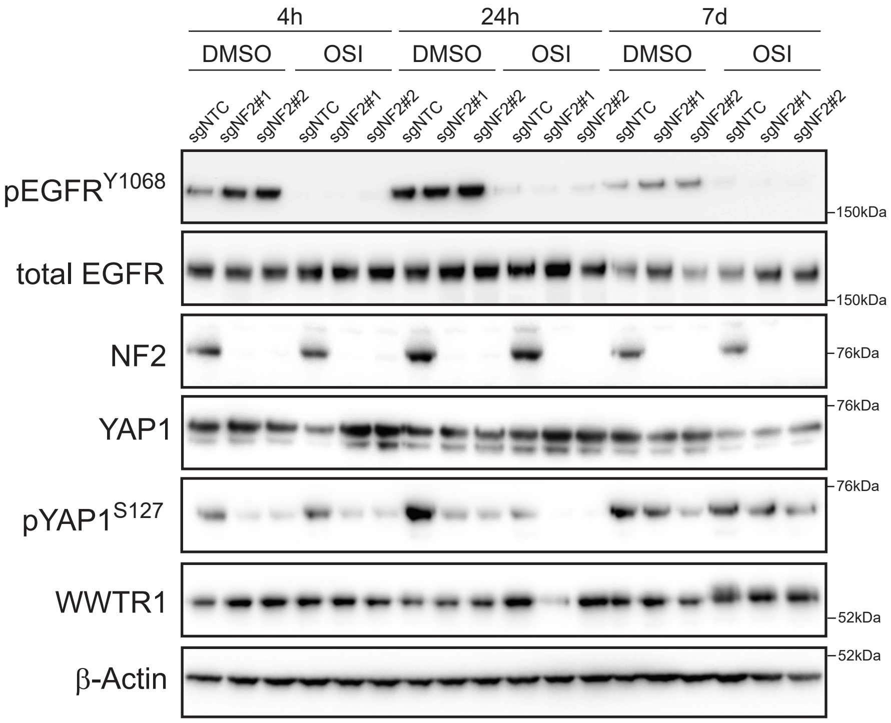

D

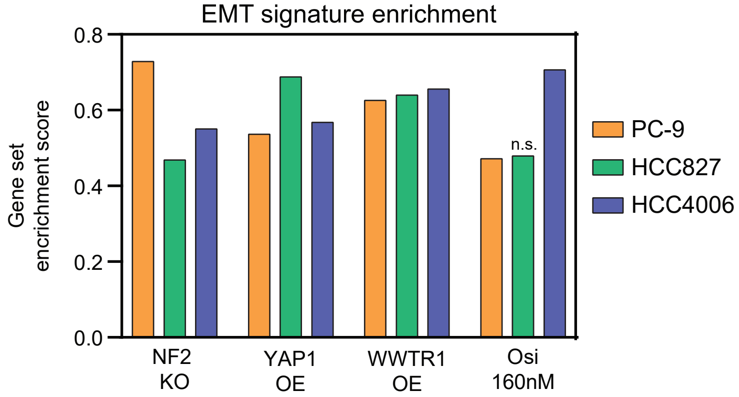

Supplementary Figure 7 (contd)

E

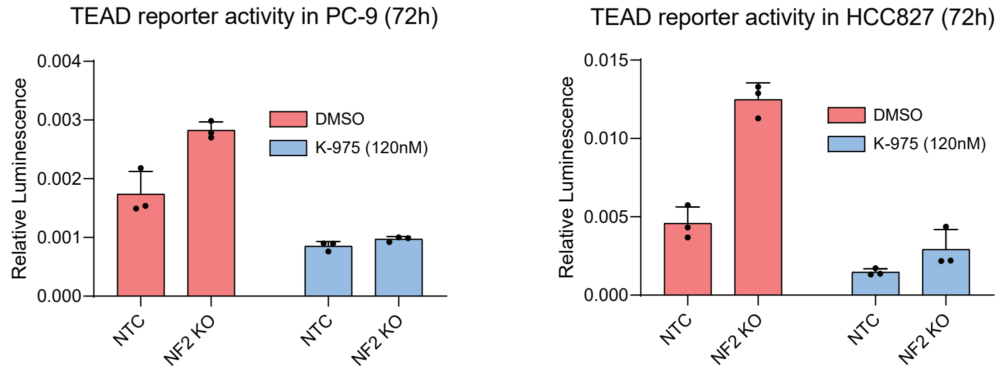

F

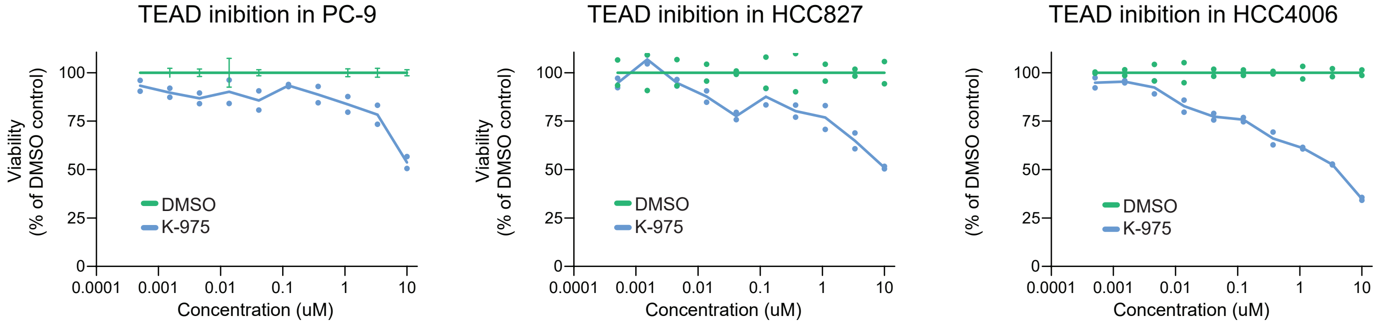

G

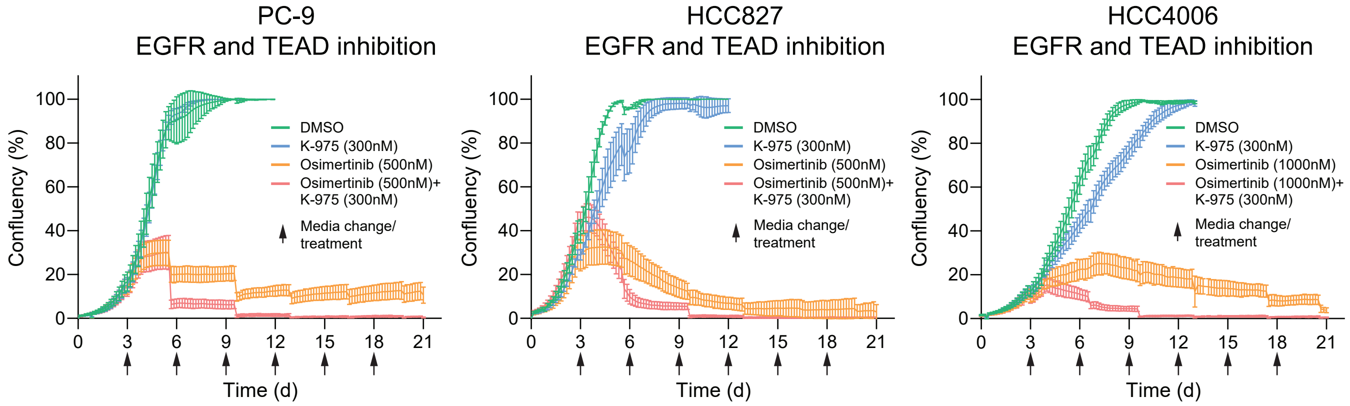

Supplementary Figure 7. (A,B) Gene set enrichment score values for canonical Hippo pathway in PC-9, HCC827 and HCC4006 cell lines following NF2 KO or overexpression of YAP1 or WWTR1. (NES= normalised enrichment score) (C) Immunoblot of isogenic PC-9 cells (NTC vs NF2 KO) and treated with Osimertinib 50nM for the indicated times. Blots probed for key components of Hippo pathway. (D) Gene set enrichment scores for EMT signature in parental PC-9, HCC827 or HCC4006 cells following treatment with osimertinib 160nM for 48 hrs. (E) NF2 KO PC-9 or HCC827 cells transduced with the TEAD reporter vector were treated with a concentration range of the TEAD inhibitors K-975 or MYF-01-037 and the effect on luciferase activity measured. (F) 6-day viability effect of K-975 or MYF-01-037 on parental PC-9, HCC827 or HCC4006 cells. (G) 21-day viability assay of osimertinib (500 or 1000nM) and K-975 (300nM) alone versus in combination in PC-9, HCC827 and HCC4006 cell lines. The higher concentration of osimertinib was used to specifically enrich for persister cells.

Supplementary Figure 8

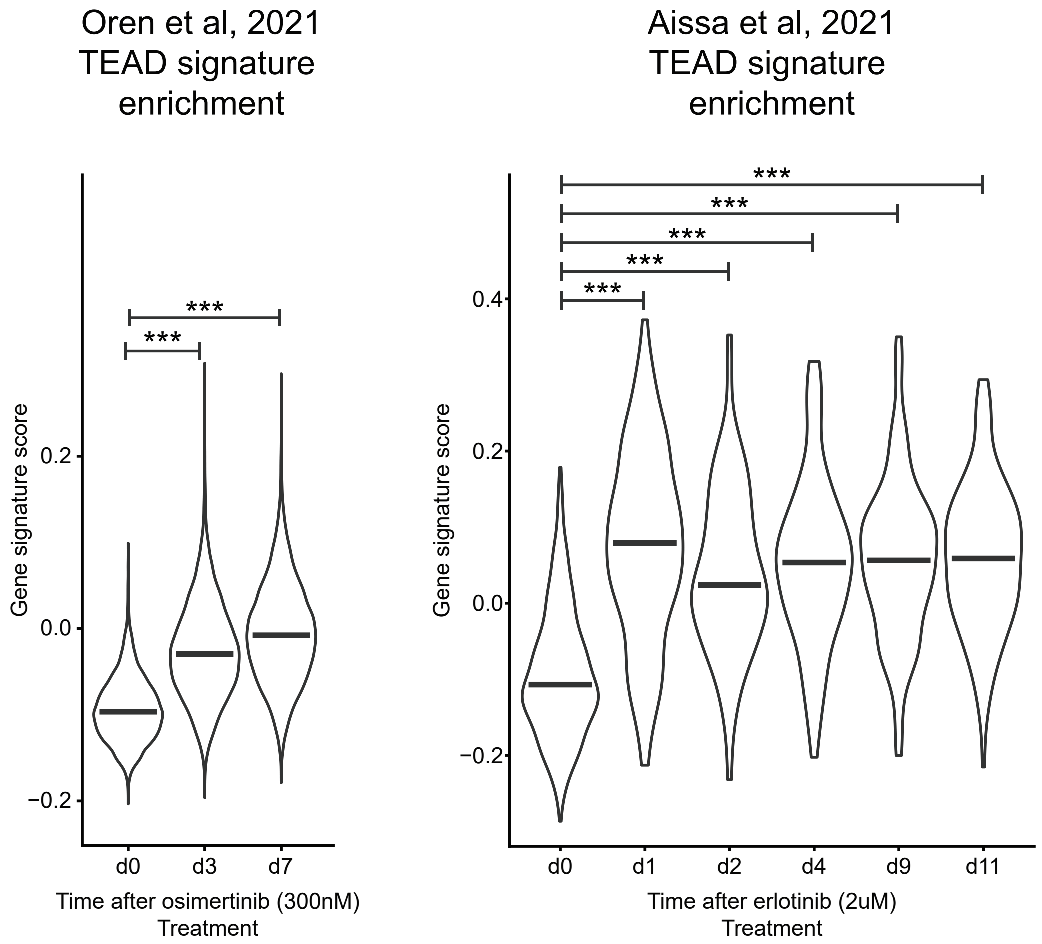

Supplementary Figure 8. Enrichment of a Hippo transcriptional signature (Zhang, 2009) from single-cell RNA-seq data in PC-9 cells following treatment with the indicated concentration of the EGFR inhibitors osimertnib or erlotinib and at the days indicated post treatment (*** p<0.001).

Supplementary Figure 9

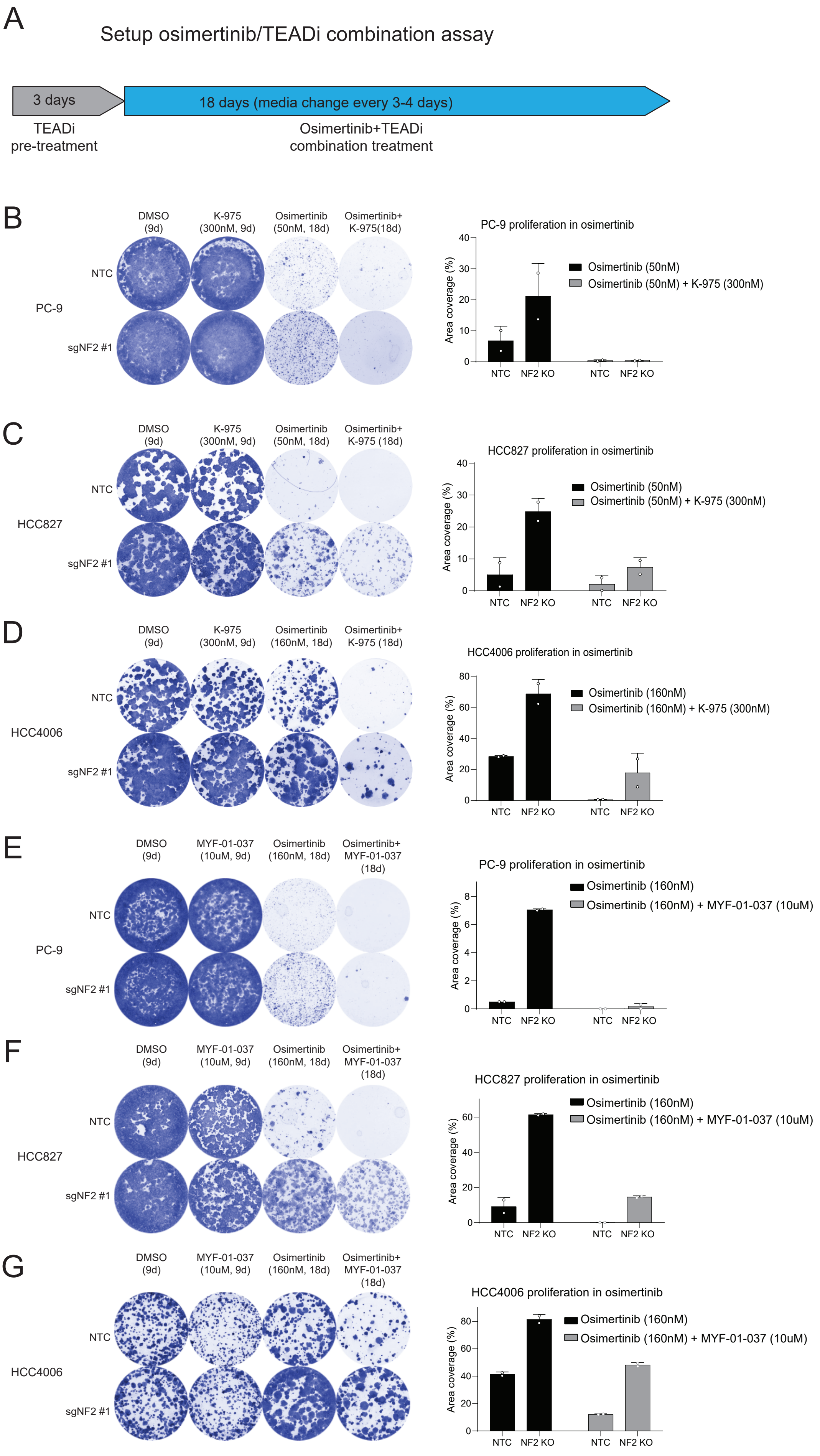

Supplementary Figure 9. (A) Schema of treatment schedule of NF2 KO vs NTC isogenics in PC-9, HCC827 and HCC4006 cell lines. (B-G) Clonogenic survival assay results at 21 days for cells pre-treated with either TEAD inhibitor (K-975 or MYF-01-037) for 3 days followed by 18 days of treatment with osimertinib, each TEAD inhibitor or the combination.

Supplementary Figure 10

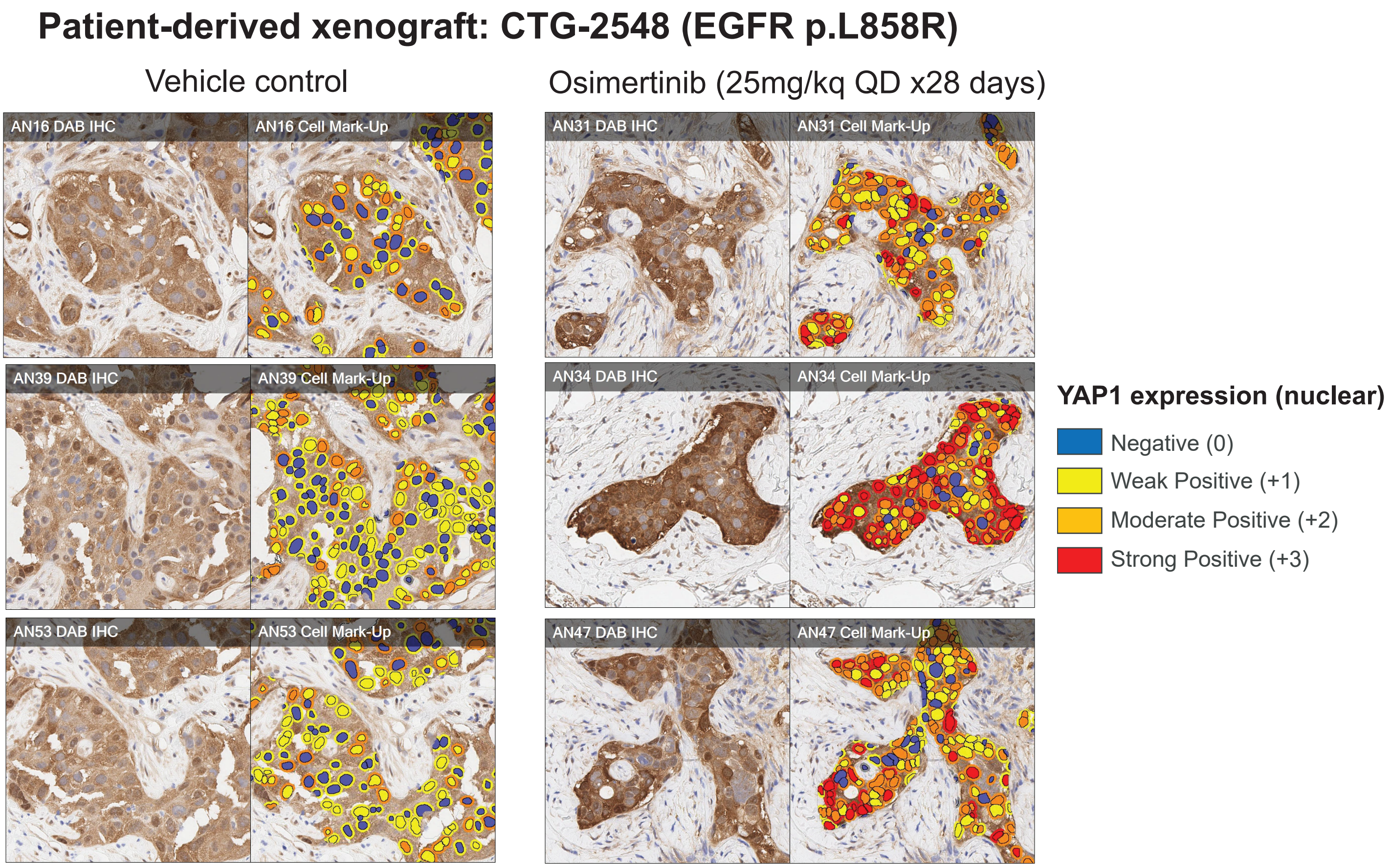

Supplementary Figure 10. (A) HC images of nuclear YAP1 expression in 3 mice engrafted with the CTG-2548 PDX and treated with a clinically relevant dose of osimertinib for 28 days (25mg/kg QDS).

Supplementary Figure 11

A

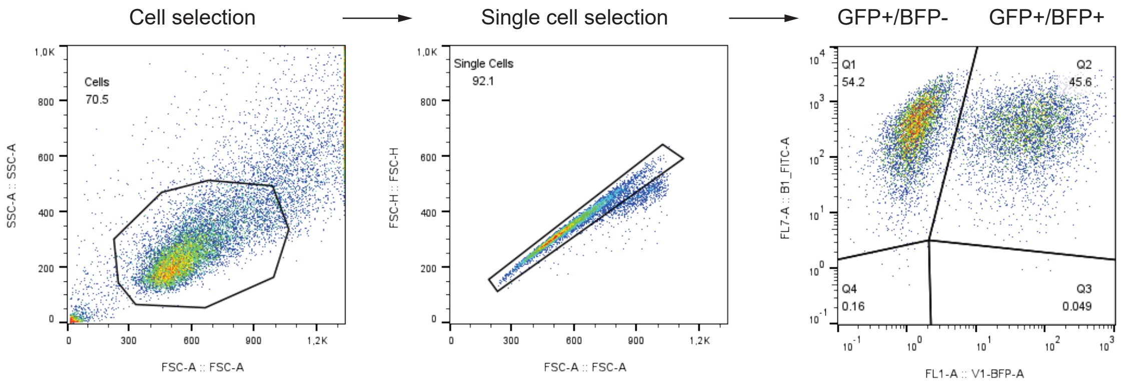

Supplementary Figure 11. (A) Flow cytometry gating strategy to discriminate GFP+/BFP- and GFP+/BFP+ cells in competition assay shown in Figure 3e. Fixed cells were selected according to FSC/SSC characteristics and singlets analysed for GFP and BFP expression.

Supplementary Figure 12

A

Figure 3B Original WB data

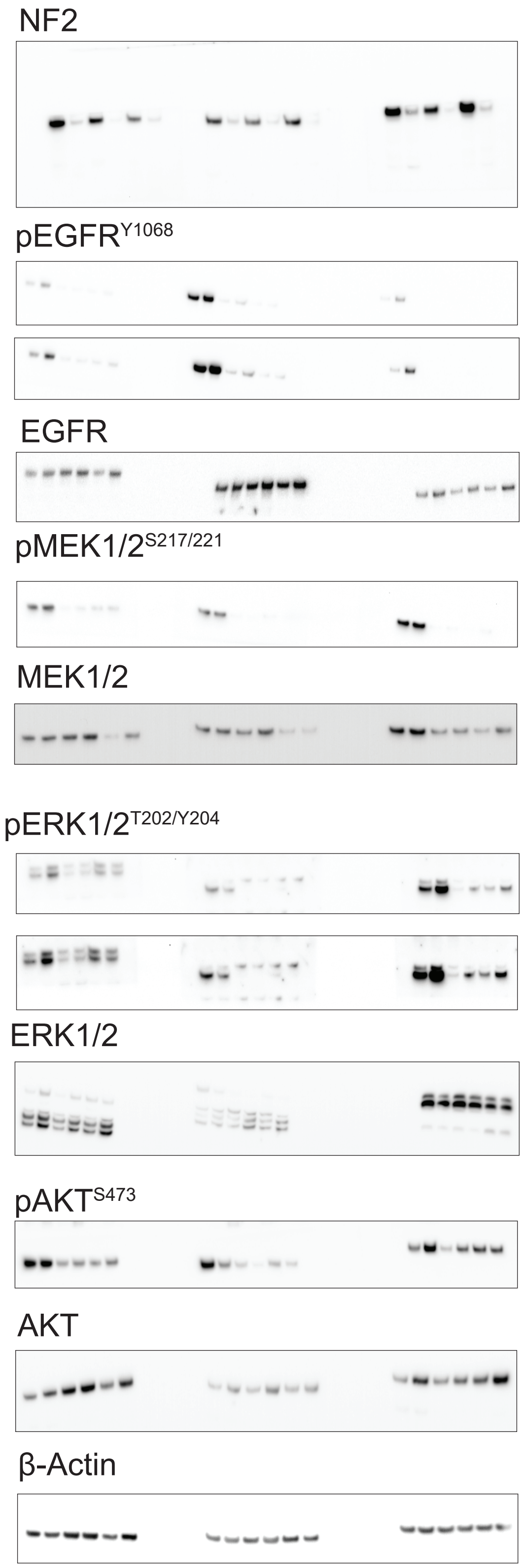

Supplementary Figure 12

B

Supplementary Figure 1J Original WB data

pEGFR^{Y1068}

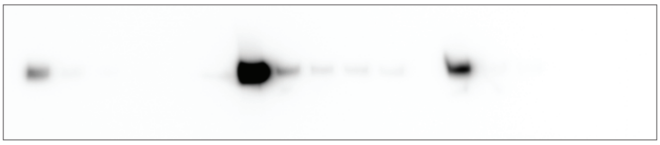

EGFR

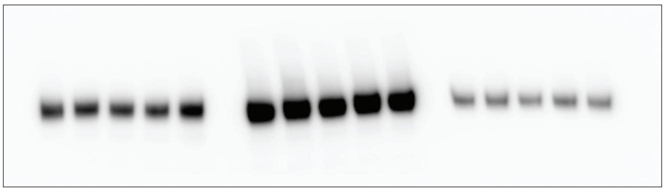

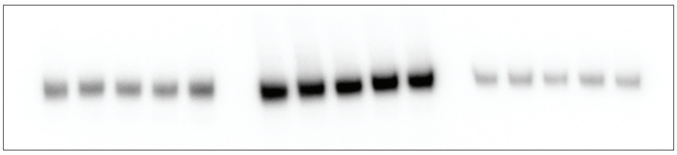

pMEK1/2$^{S217/221}$

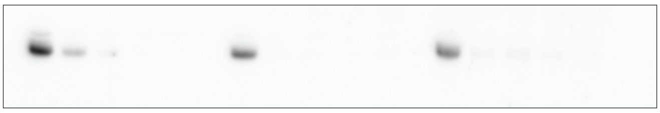

MEK1/2

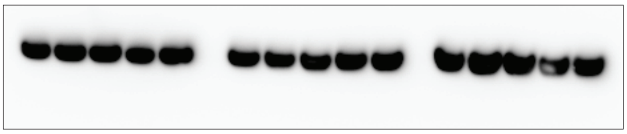

β-Actin

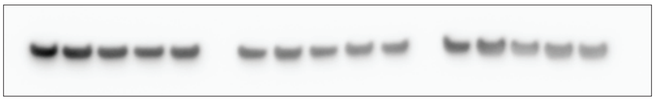

Supplementary Figure 12C

Supplementary Figure 4A Original WB data

PC-9 CSK

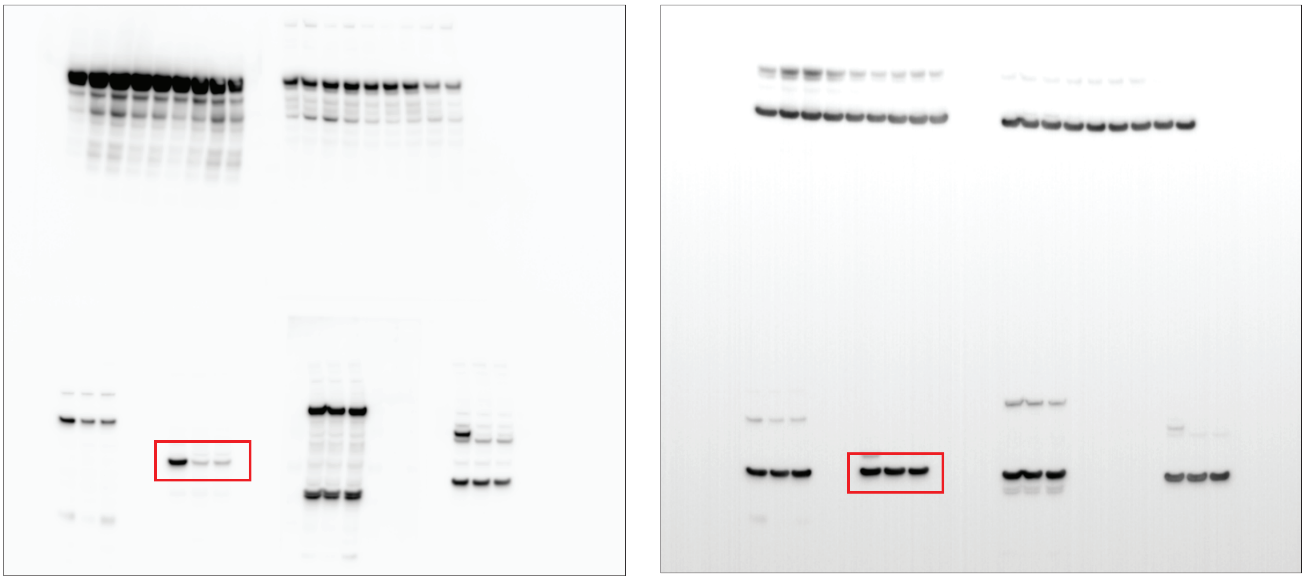

PC-9 MED24

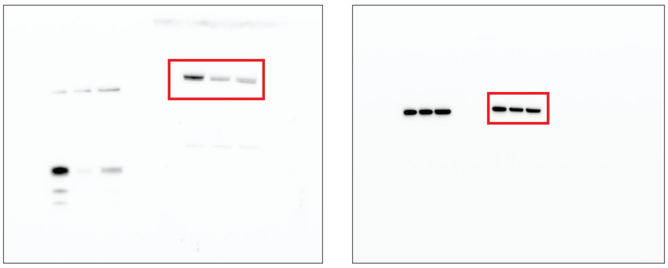

PC-9 NF1

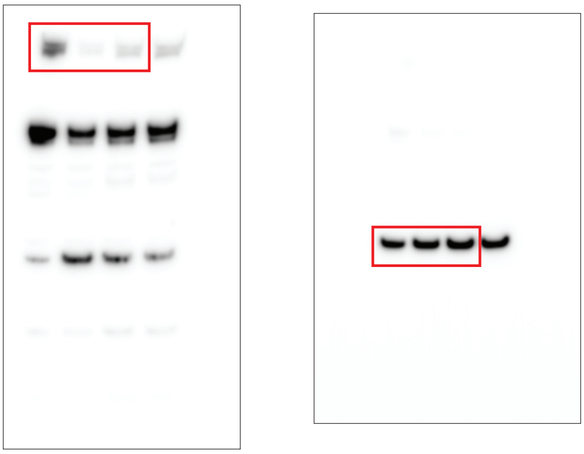

PC-9 KCTD5

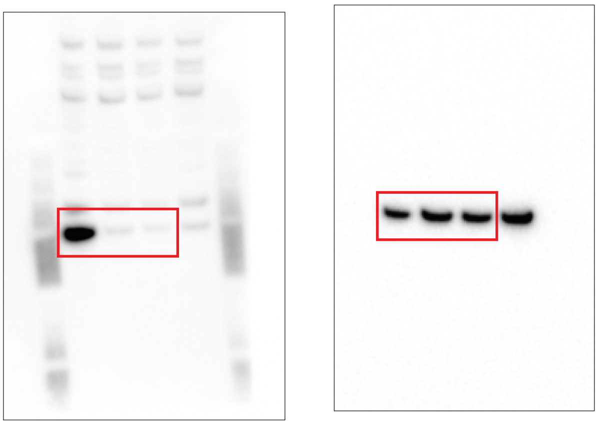

PC-9 PTEN

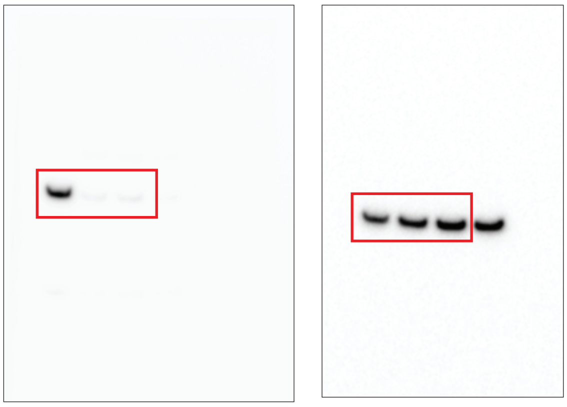

Supplementary Figure 12C

Supplementary Figure 4A Original WB data

HCC827

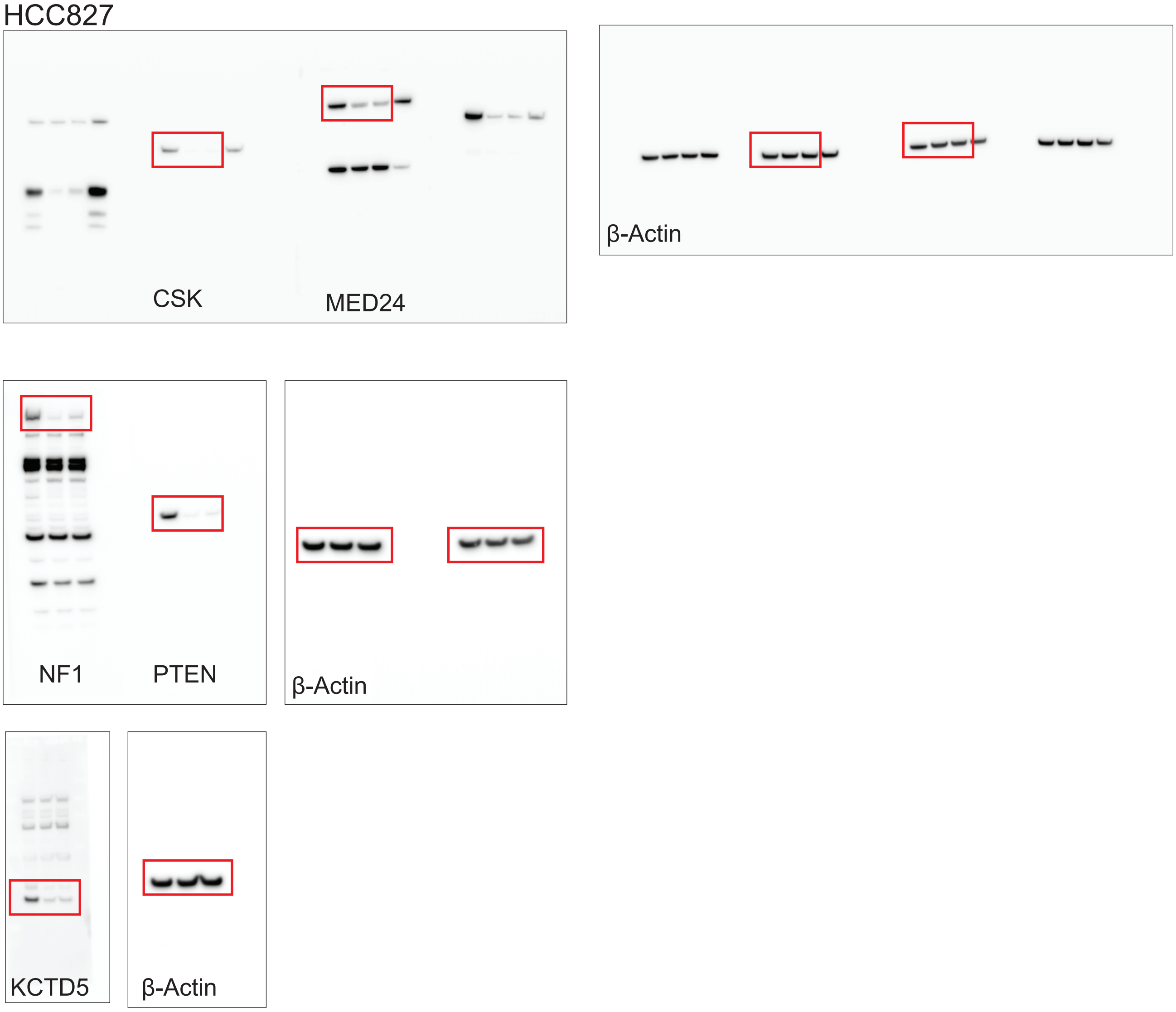

Supplementary Figure 12C

Supplementary Figure 1C Original WB data

HCC4006

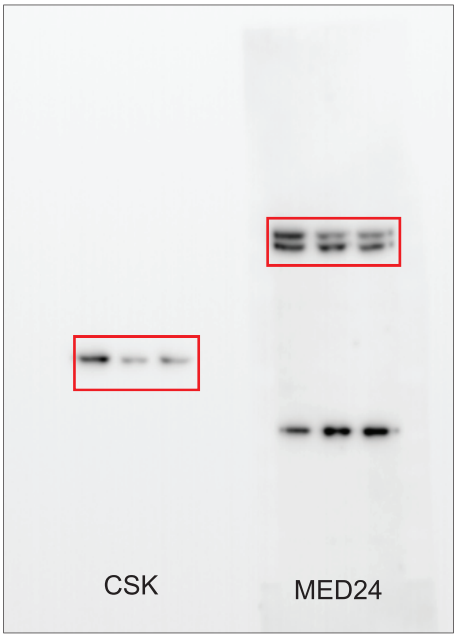

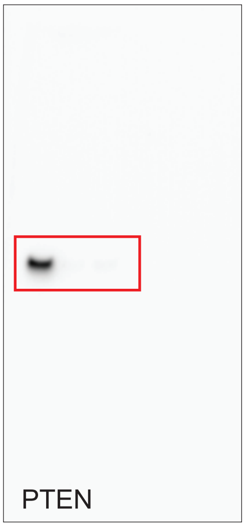

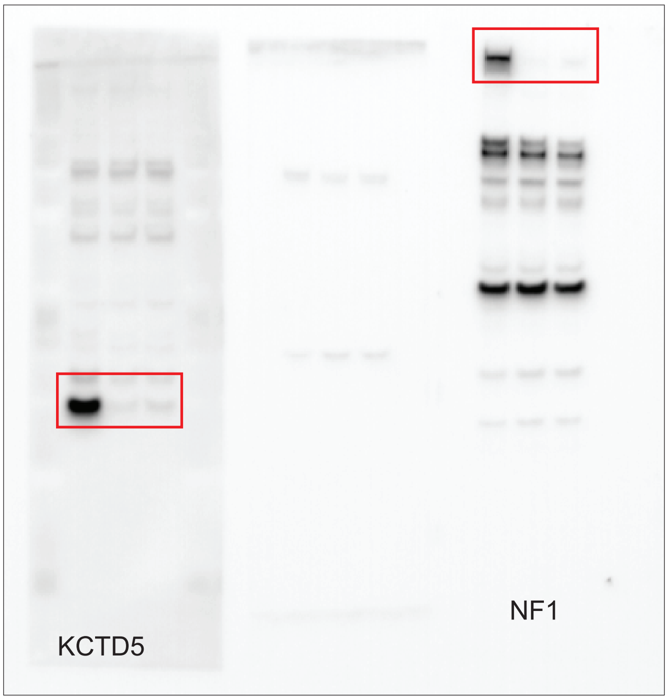

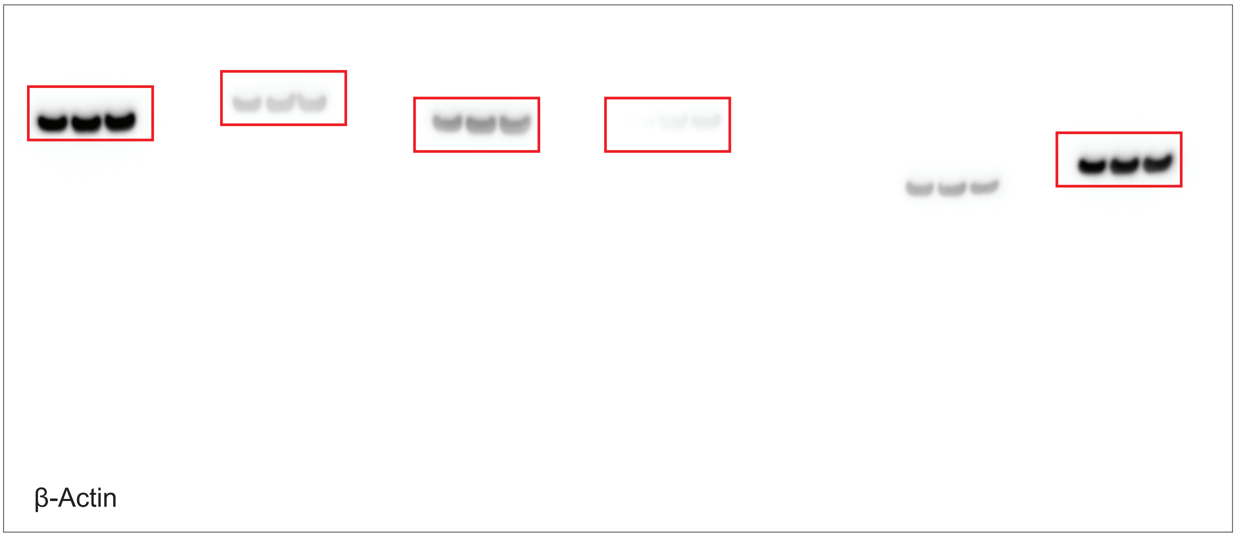

Supplementary Figure 12D

Supplementary Figure 4C Original WB data

MET

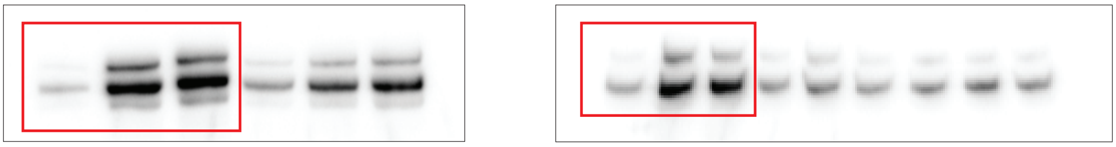

β-Actin

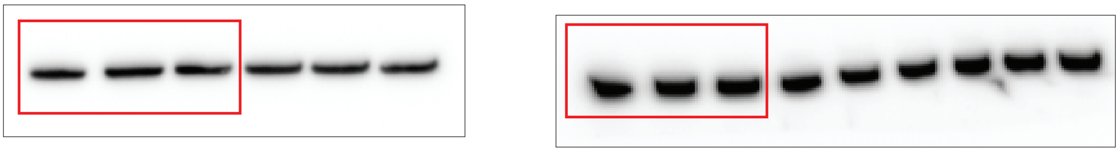

Supplementary Figure 12E

Supplementary Figure 5A Original WB data

PC-9 NF2

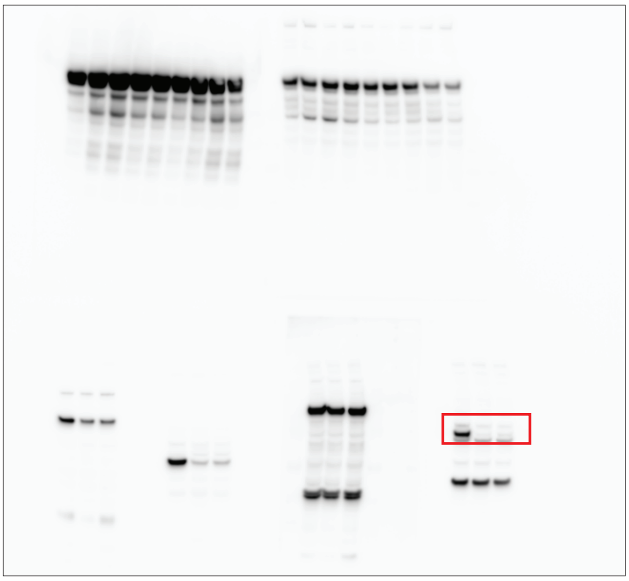

PC-9 β-Actin

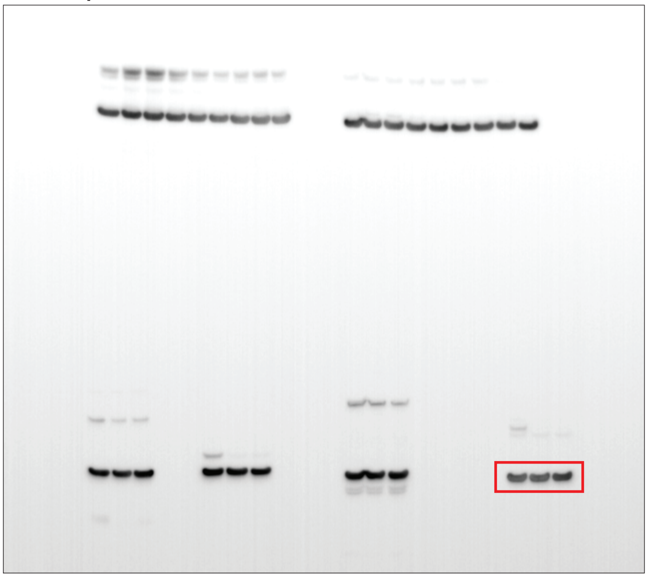

HCC827 NF2

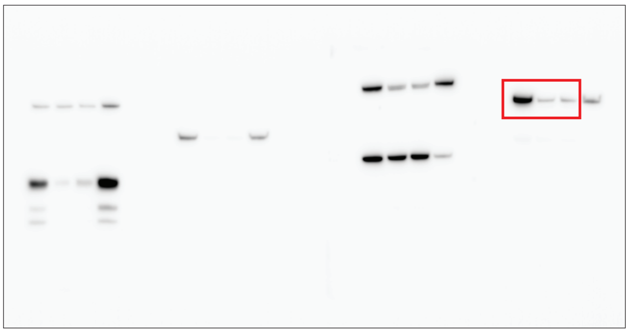

HCC827 β-Actin

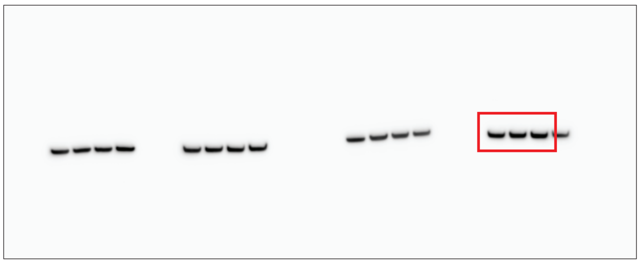

HCC4006 NF2

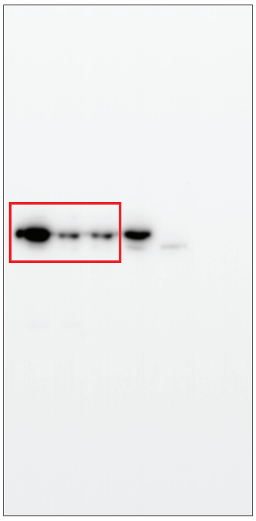

HCC4006 β-Actin

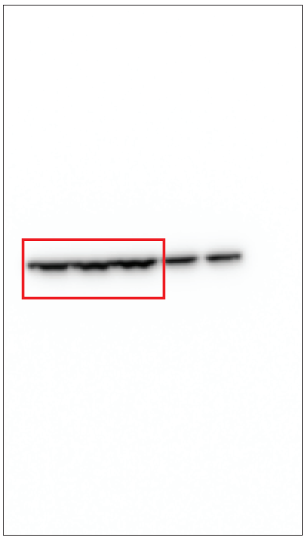

Supplementary Figure 12F

Supplementary Figure 5B Original WB data

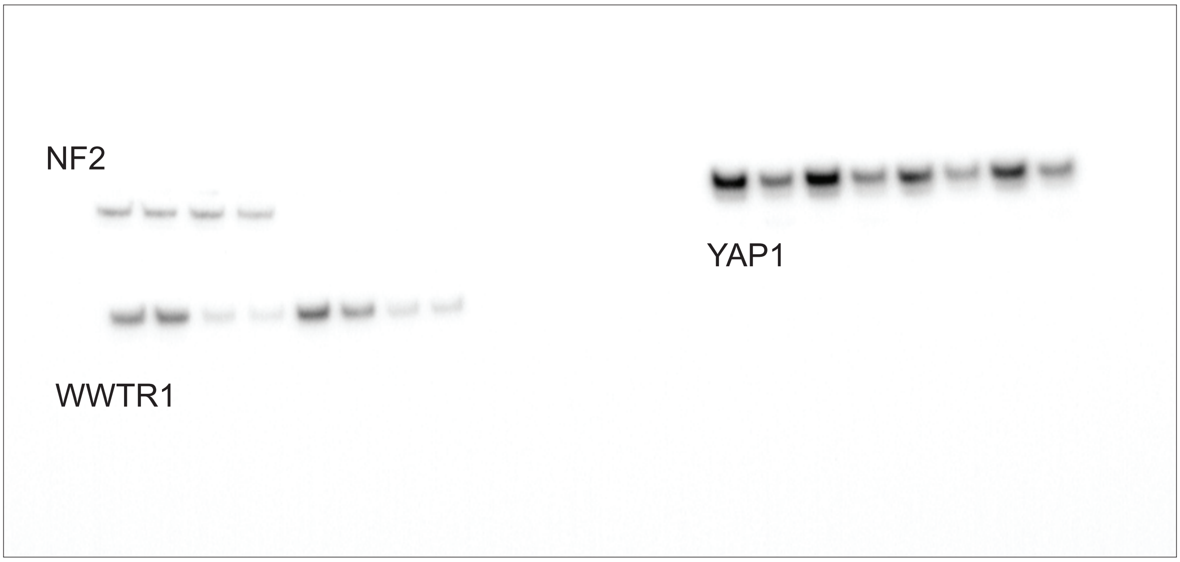

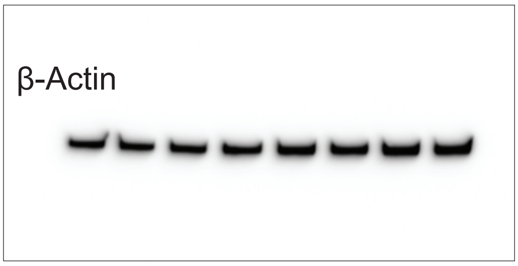

Supplementary Figure 12G

Supplementary Figure 6A Original WB data

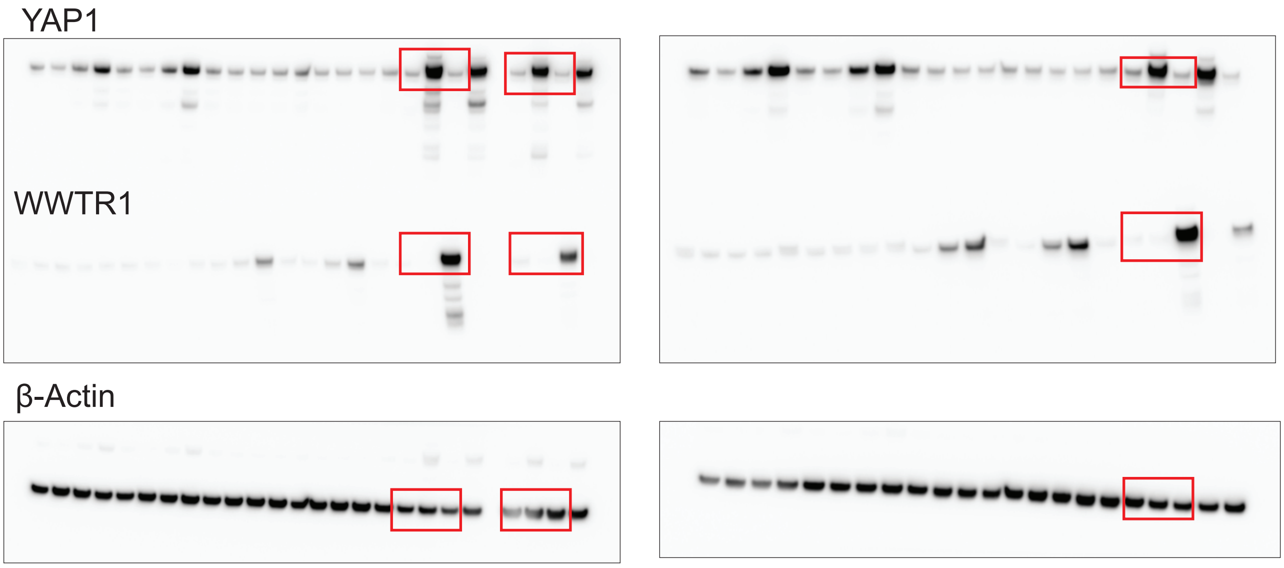

H

Supplementary Figure 6C Original WB data

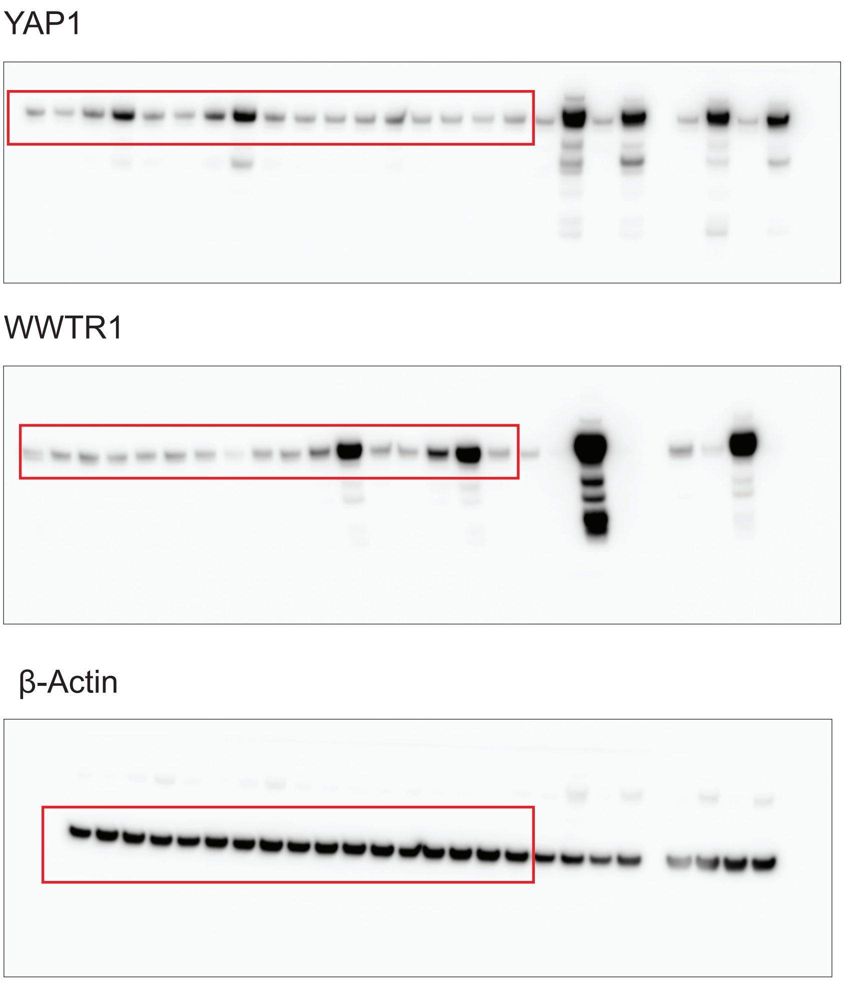

Supplementary Figure 12H

Supplementary Figure 7C Original WB data

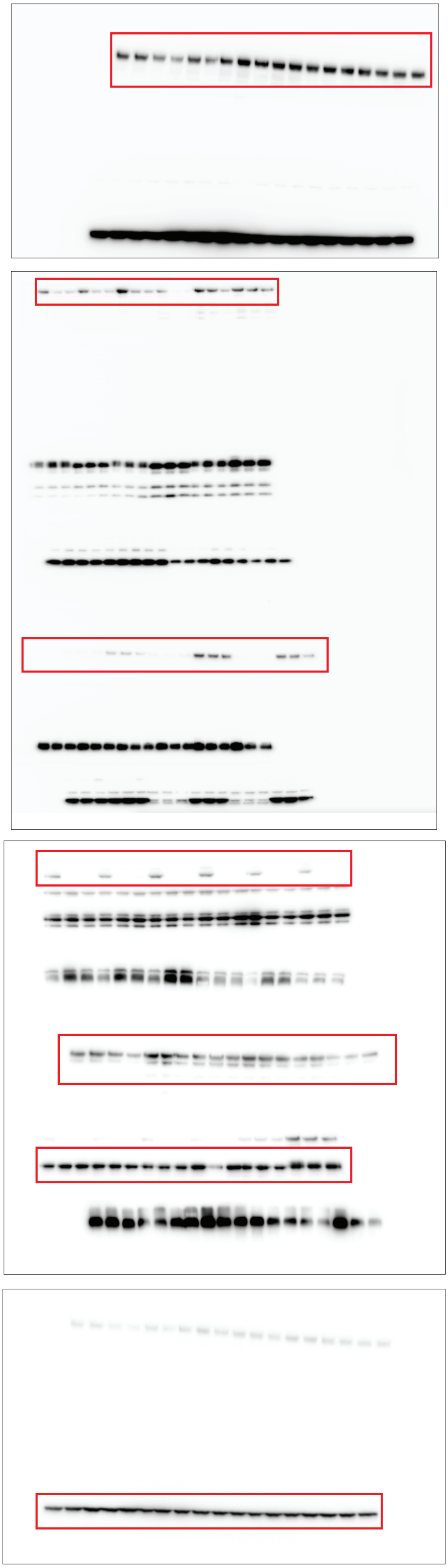

total EGFR

pYAP1^{S127}

pEGFR^{Y1068}

NF2

YAP1

WWTR1

β-Actin

Supplementary Figure 12I

Supplementary Figure 1C Original WB data

EGFR

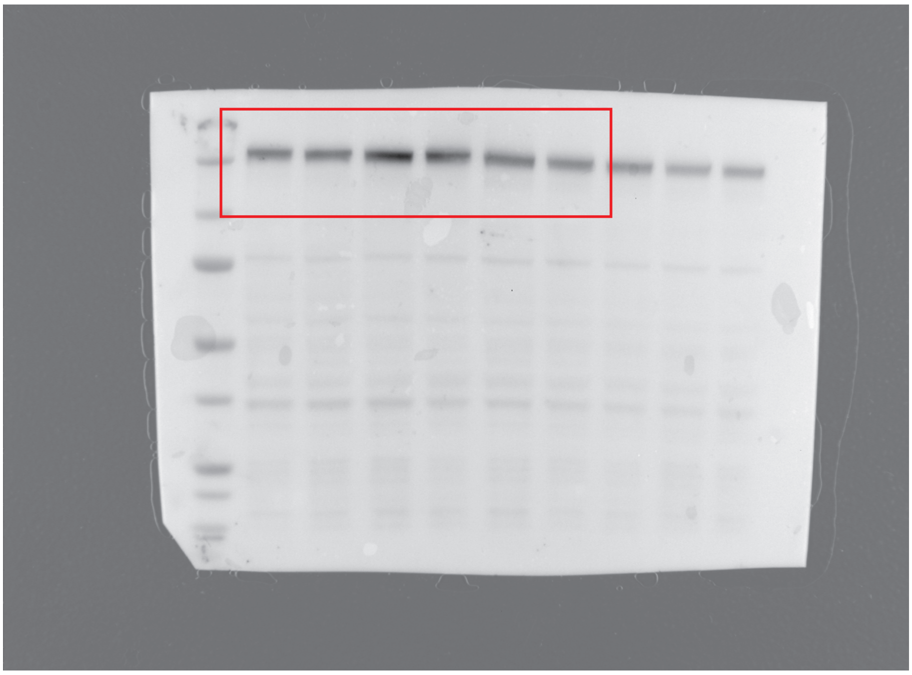

pEGFR

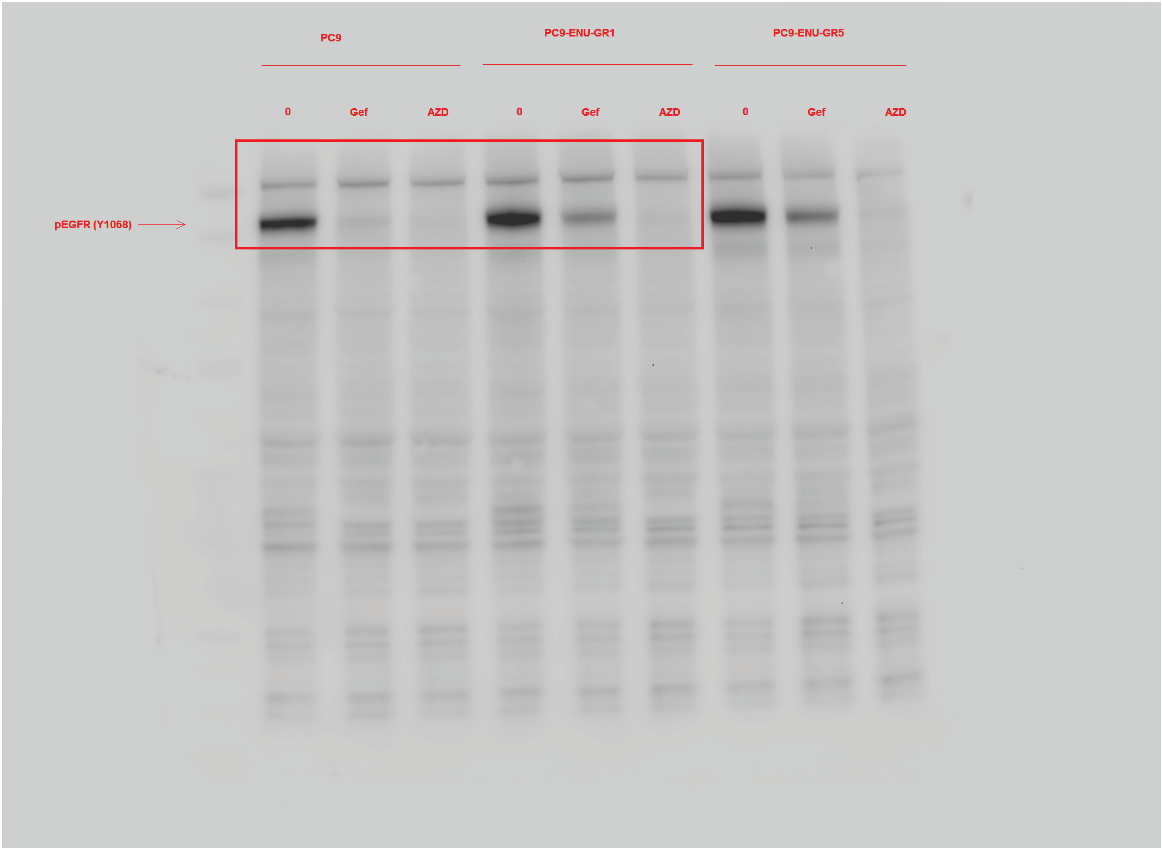

Supplementary Figure 12I

Supplementary Figure 1C Original WB data

PC9 / PC9 T790M pAKT pERK AKT ERK

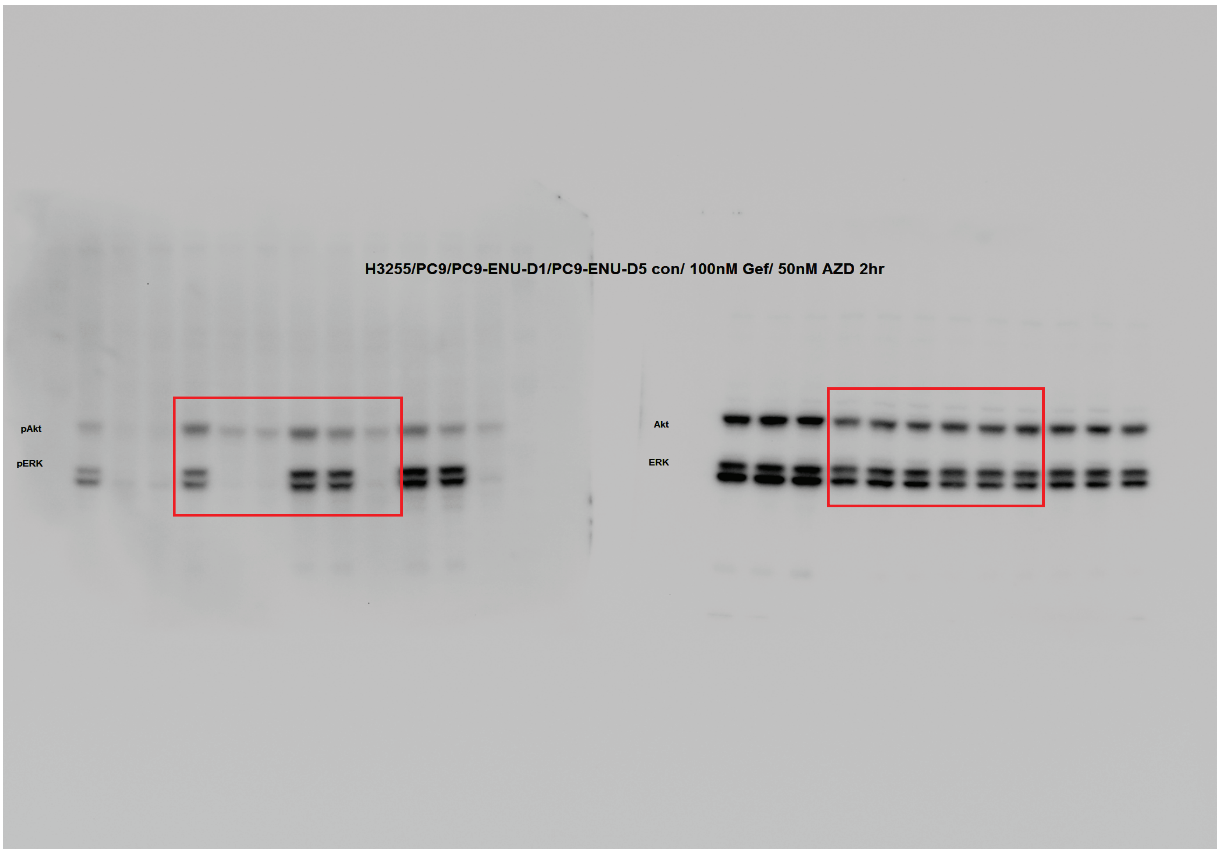

HCC827 / HCC827 T790M pEGFR EGFR

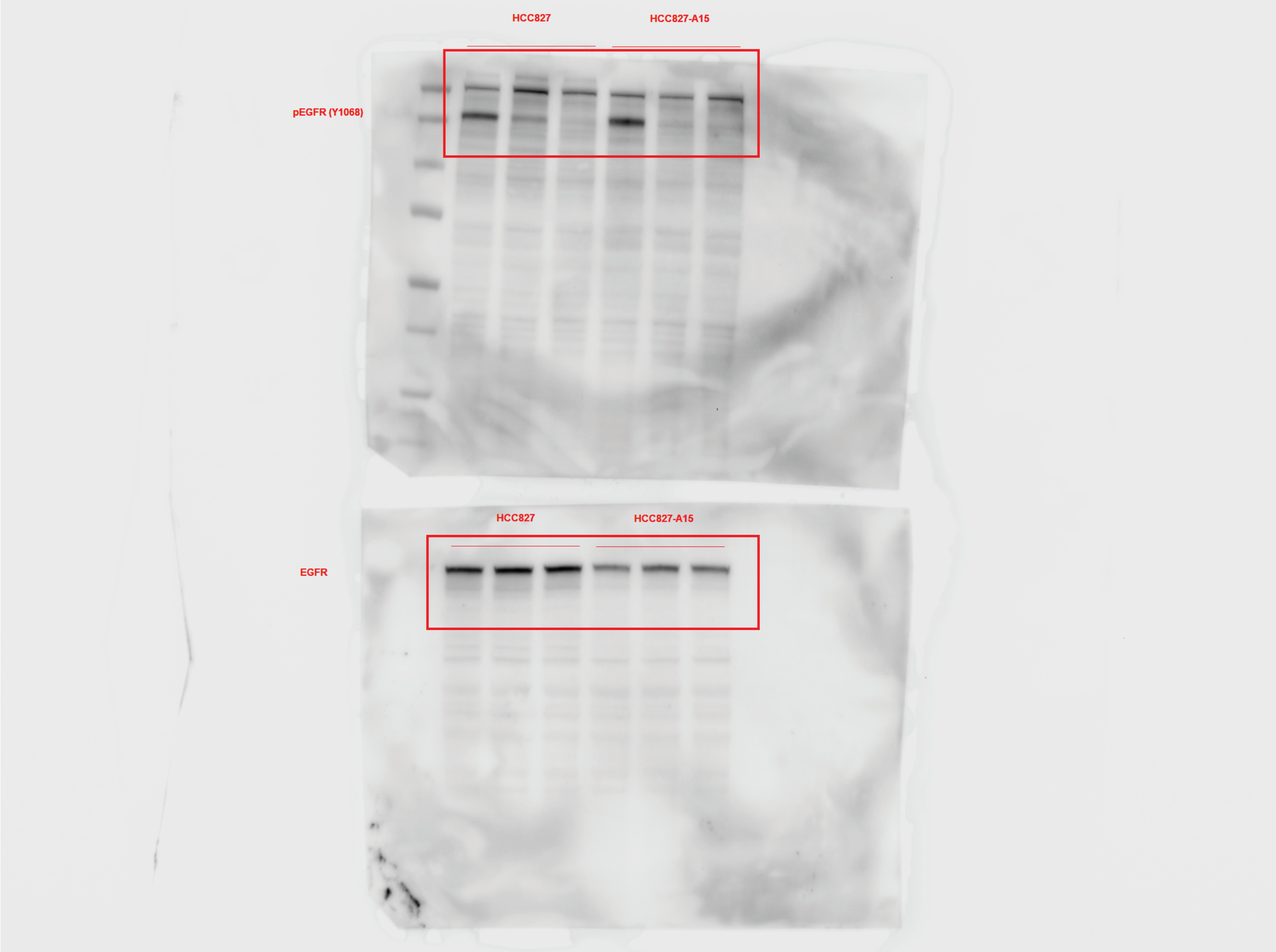

Supplementary Figure 12I

Supplementary Figure 1C Original WB data

HCC827 / HCC827 T790M pERK pAKT

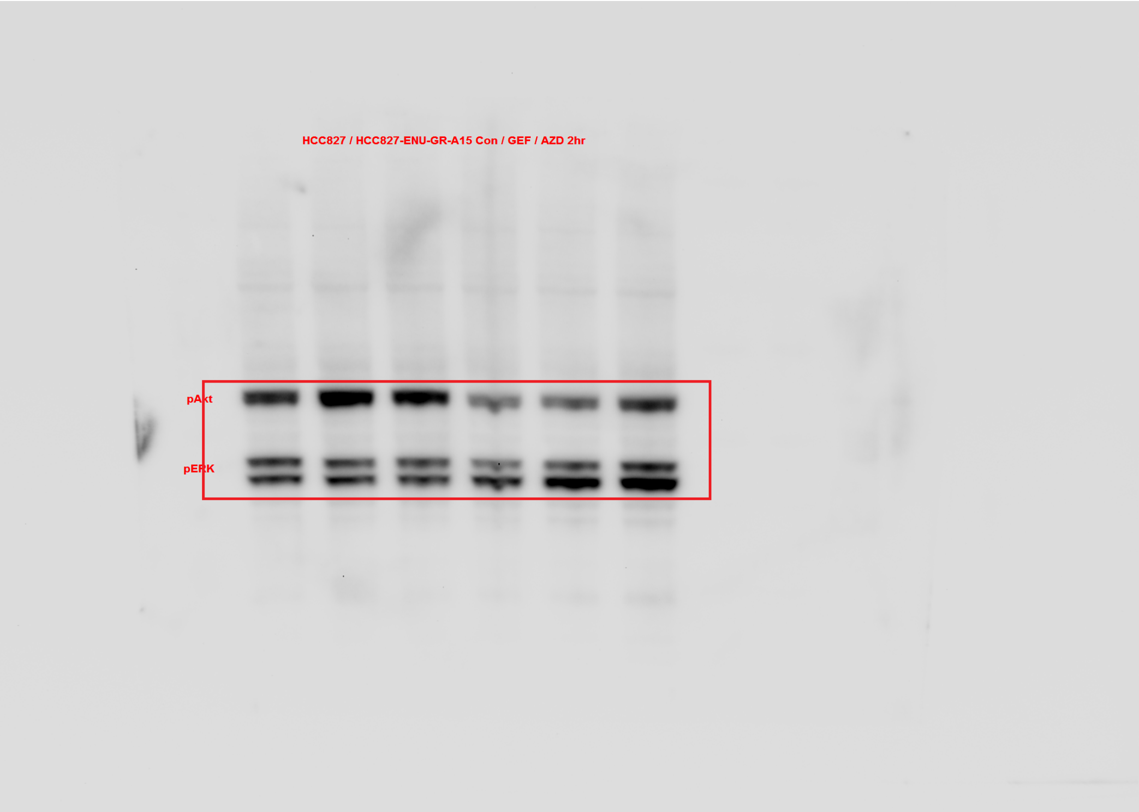

HCC827 / HCC827 T790M ERK AKT

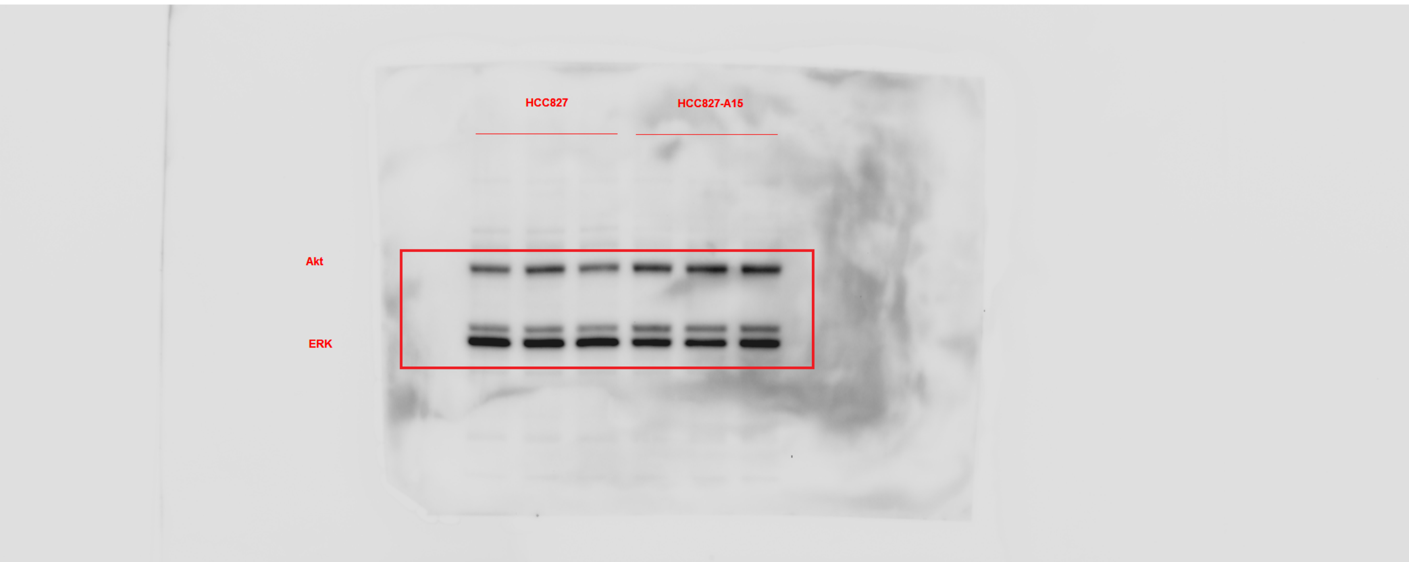

Supplementary Figure 12I

Supplementary Figure 1C Original WB data

HCC827 / HCC827 T790M tubulin

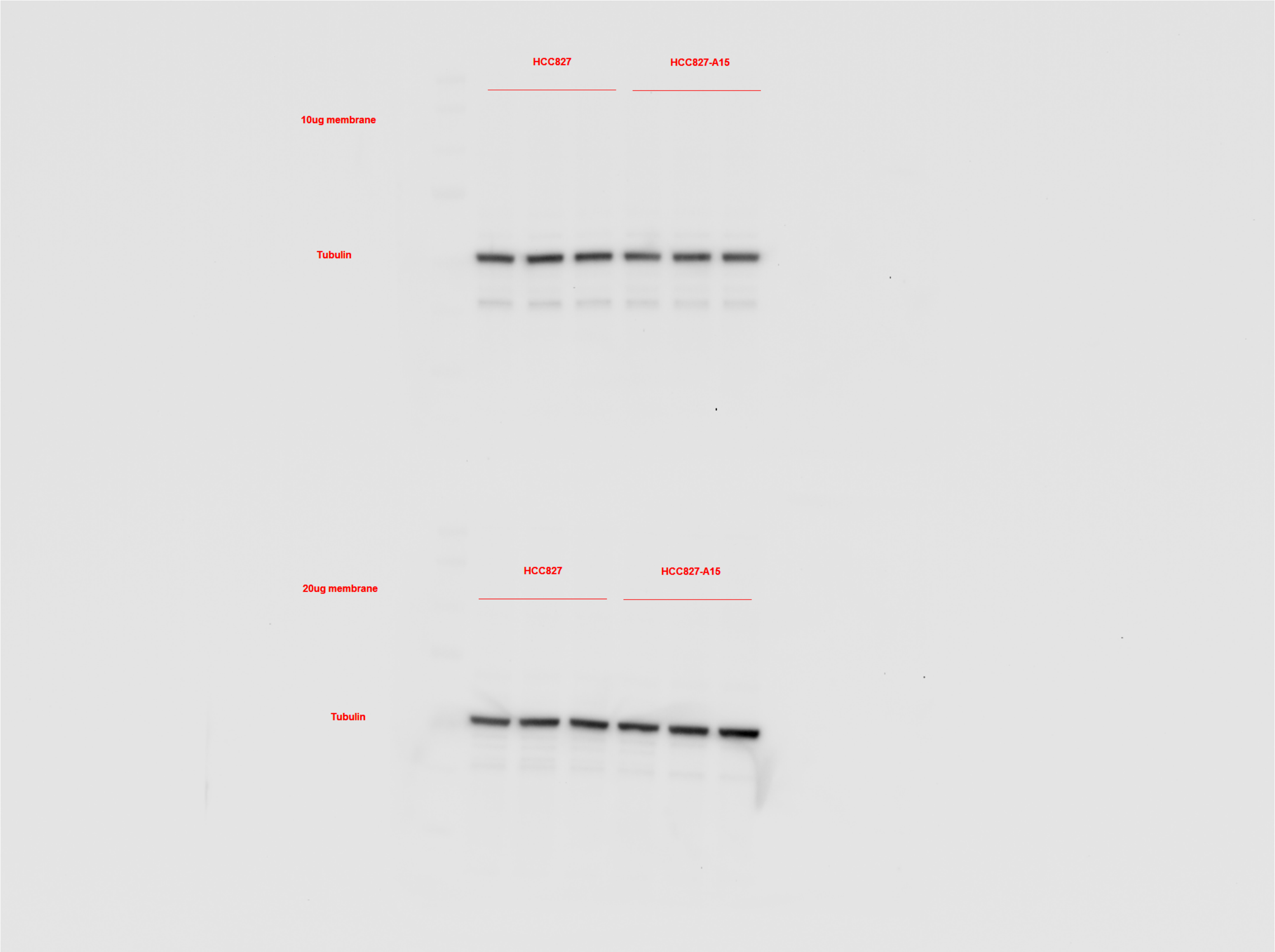

Supplementary Information

AstraZeneca Cancer Research Horizons Functional Genomics Centre (FGC)

Douglas Ross-Thriepland[3], David Walter[6], Sebastian Lukasiak[3], Angelos Papadopoulos[3], Amna Shah[1], Josh Tweedy[1], Rebecca McRae[1], Khamal Ampah[1], Ashutosh Trehan[1], Maryam Ghaderi[1], Lu Li[1], Toby Gurran[1], Carlos Company[1], Daniel Barrell[1], Khalid Saeed[6], Jennifer Hillis[6], Adam Spruce[6], Mel Lad[6], Abigail Shurr[6], Dave Kim[6], Curtis Hart[6], Rebecca England[6], Nikhil Gupta[6], Altea Targa[6], Nadana Shankar[6], Malwina Prater[6], Andy Sayer[6], Alex Kalinka[6], Marica Gaspari[6], Greg Hannon[7], Ultan McDermott[1].

Affiliations:
1. Oncology R&D, AstraZeneca, 1 Francis Crick Avenue, Cambridge, UK CB2 0RE
3. Discovery Sciences, BioPharmaceuticals R&D, AstraZeneca, 1 Francis Crick Avenue, Cambridge, UK CB2 0RE
6. Cancer Research Horizons, 2 Redman Place, London, UK E20 1JQ
7. CRUK Cambridge Institute, Li Ka Shing Centre, University of Cambridge, Cambridge, UK CB2 0RE
