# Supplementary material for: Genome-wide CRISPR screens identify the YAP/TEAD axis as a driver of persister cells in EGFR mutant lung cancer
Source: Commun Biol. 2024 Apr 24;7:497. doi: 10.1038/s42003-024-06190-w (PMC11043391; doi:10.1038/s42003-024-06190-w)
Supplement: Supplementary file 3 — Description of Additional Supplementary Files [file 42003_2024_6190_MOESM3_ESM.pdf]

Description of Additional Supplementary Files

File name: Supplementary Data 1
Description: Significantly enriched gefitinib or osimertinib resistance genes in *EGFR* mutant lung cancer.

File name: Supplementary Data 2
Description: Genes ranked for enrichment: MAGecK analysis of gefitinib vs control PC-9 cells following transduction with a genome-wide CRISPRn or CRISPRa library.

File name: Supplementary Data 3
Description: Genes ranked for enrichment: MAGecK analysis of osimertinib vs control PC-9 cells following transduction with a genome-wide CRISPRn or CRISPRa library.

File name: Supplementary Data 4
Description: Genes ranked for enrichment: MAGecK analysis of gefitinib vs control HCC827 cells following transduction with a genome-wide CRISPRn or CRISPRa library.

File name: Supplementary Data 5
Description: Genes ranked for enrichment: MAGecK analysis of osimertinib vs control HCC827 cells following transduction with a genome-wide CRISPRn or CRISPRa library.

File name: Supplementary Data 6
Description: Genes ranked for enrichment: MAGecK analysis of osimertinib vs control PC-9 T790M cells following transduction with a genome-wide CRISPRn or CRISPRa library.

File name: Supplementary Data 7
Description: Genes ranked for enrichment: MAGecK analysis of osimertinib vs control HCC827 T790M cells following transduction with a genome-wide CRISPRn or CRISPRa library.

File name: Supplementary Data 8
Description: Arrayed CRISPR screen in PC-9 cells of resistance genes with high content microscopy quantification of PI3K/MAPK/mTOR and Hippo pathway components following osimertinib treatment.

File name: Supplementary Data 9
Description: Arrayed CRISPR screen in HCC4006 cells of resistance genes with high content microscopy quantification of PI3K/MAPK/mTOR and Hippo pathway components following osimertinib treatment.

File name: Supplementary Data 10
Description: Arrayed CRISPR screen in HCC827 cells of resistance genes with high content microscopy quantification of PI3K/MAPK/mTOR and Hippo pathway components following osimertinib treatment.

File name: Supplementary Data 11
Description: Significantly enriched sensitizing genes to EGFR kinase inhibition in *EGFR* mutant lung cancer.

File name: Supplementary Data 12
Description: Differentially expressed genes detected in isogenic models (NF2 KO, WWTR1 OE, YAP1 OE or osimertinib treatment) of PC-9, HCC827 and HCC4006 cell lines.

File name: Supplementary Data 13
Description: Transcription factor (TF) activity scores in PC-9, HCC827 and HCC4006 cell lines following knockout (KO) or overexpression (OE) of the Hippo resistance genes NF2, WWTR1 and YAP1.

File name: Supplementary Data 14
Description: Gene set enrichment analyses of Molecular Signatures Database Hallmarks genesets detected in (a) isogenic models (NF2 KO, YAP1 OE, WWTR1 OE) versus parental PC-9, HCC827 and HCC4006 cell lines and (b) Osimertinib treated vs untreated cells at 24 hours.

File name: Supplementary Data 15
Description: Nuclear YAP1 and WWTR1 expression in osimertinib sensitive or resistant *EGFR* mutant lung cancer PDX models.

File name: Supplementary Data 16
Description: Q-PCR mRNA gene expression of canonical Hippo transcripts CTGF, CYR61 and AMOTL2 in *EGFR* mutant 2D cell lines and 3D organoid models following osimertinib treatment.

File name: Supplementary Data 17
Description: Guide RNA targeting sequences.

File name: Supplementary Data 18
Description: JASPAR and TRANSFAC database transcription factors (TF) used to infer TF activity from RNA-seq datasets.
