# Supplementary material for: Genome-wide CRISPR screens identify the YAP/TEAD axis as a driver of persister cells in EGFR mutant lung cancer
Source: Commun Biol. 2024 Apr 24;7:497. doi: 10.1038/s42003-024-06190-w (PMC11043391; doi:10.1038/s42003-024-06190-w)
Supplement: Supplementary file 5 — Reporting Summary [file 42003_2024_6190_MOESM5_ESM.pdf]

nature portfolio

Corresponding author(s): Ultan McDermott

Last updated by author(s): Feb 27, 2024

Reporting Summary

Nature Portfolio wishes to improve the reproducibility of the work that we publish. This form provides structure for consistency and transparency in reporting. For further information on Nature Portfolio policies, see our Editorial Policies and the Editorial Policy Checklist.

Statistics

For all statistical analyses, confirm that the following items are present in the figure legend, table legend, main text, or Methods section.

n/a	Confirmed	
☐	☒	The exact sample size (*n*) for each experimental group/condition, given as a discrete number and unit of measurement
☐	☒	A statement on whether measurements were taken from distinct samples or whether the same sample was measured repeatedly
☐	☒	The statistical test(s) used AND whether they are one- or two-sided<br>*Only common tests should be described solely by name; describe more complex techniques in the Methods section.*
☒	☐	A description of all covariates tested
☒	☐	A description of any assumptions or corrections, such as tests of normality and adjustment for multiple comparisons
☒	☐	A full description of the statistical parameters including central tendency (e.g. means) or other basic estimates (e.g. regression coefficient) AND variation (e.g. standard deviation) or associated estimates of uncertainty (e.g. confidence intervals)
☒	☐	For null hypothesis testing, the test statistic (e.g. *F*, *t*, *r*) with confidence intervals, effect sizes, degrees of freedom and *P* value noted<br>*Give P values as exact values whenever suitable.*
☒	☐	For Bayesian analysis, information on the choice of priors and Markov chain Monte Carlo settings
☒	☐	For hierarchical and complex designs, identification of the appropriate level for tests and full reporting of outcomes
☒	☐	Estimates of effect sizes (e.g. Cohen's *d*, Pearson's *r*), indicating how they were calculated

Our web collection on statistics for biologists contains articles on many of the points above.

Software and code

Policy information about availability of computer code

Data collection	*Provide a description of all commercial, open source and custom code used to collect the data in this study, specifying the version used OR state that no software was used.*
Data analysis	RNA-sequencing: The STAR software (v2.6.1d) was used to align reads to genome build hg38 (https://github.com/alexdobin/STAR/releases). Data was analyzed by ROSALIND® (https://rosalind.bio/), with a HyperScale architecture developed by ROSALIND, Inc. (San Diego, CA).

For manuscripts utilizing custom algorithms or software that are central to the research but not yet described in published literature, software must be made available to editors and reviewers. We strongly encourage code deposition in a community repository (e.g. GitHub). See the Nature Portfolio guidelines for submitting code & software for further information.

Data

Policy information about availability of data

All manuscripts must include a data availability statement. This statement should provide the following information, where applicable:

- Accession codes, unique identifiers, or web links for publicly available datasets
- A description of any restrictions on data availability
- For clinical datasets or third party data, please ensure that the statement adheres to our policy

The read counts tables for all CRISPR datasets and also the complete MAGeCK gene summary files for each experiment are available to download: https://az.box.com/s/bgxjw7l7pjkbektqw59h84iiz1anpjij.

Raw FASTQ files from RNA-sequencing analyses in the paper are available in ArrayExpress in the study 'EGFR mutant human non-small cell lung cancer cell lines PC9, HCC827, HCC4006 after NF2 knockout, YAP1 or WWTR1 overexpression or osimertinib treatment.' under accession E-MTAB-13831.
All other data are available from the corresponding author (or other sources, as applicable) on reasonable request.

Research involving human participants, their data, or biological material

Policy information about studies with human participants or human data. See also policy information about sex, gender (identity/presentation), and sexual orientation and race, ethnicity and racism.

Reporting on sex and gender	Reporting information on sex and gender is not of relevance for the data presented.
Reporting on race, ethnicity, or other socially relevant groupings	Reporting information on race, ethnicity, or other socially relevant groupings is not of relevance for the data presented.
Population characteristics	Not applicable
Recruitment	Not applicable
Ethics oversight	Not applicable

Note that full information on the approval of the study protocol must also be provided in the manuscript.

Field-specific reporting

Please select the one below that is the best fit for your research. If you are not sure, read the appropriate sections before making your selection.

☒ Life sciences ☐ Behavioural & social sciences ☐ Ecological, evolutionary & environmental sciences

For a reference copy of the document with all sections, see nature.com/documents/nr-reporting-summary-flat.pdf

Life sciences study design

All studies must disclose on these points even when the disclosure is negative.

Sample size	Minimal sample size of data presented is at least two biological replicates.
Data exclusions	Data has not been excluded.
Replication	Measures were taken at least as biological replicates.
Randomization	No randomization has been applied.
Blinding	No blinding has been applied.

Reporting for specific materials, systems and methods

We require information from authors about some types of materials, experimental systems and methods used in many studies. Here, indicate whether each material, system or method listed is relevant to your study. If you are not sure if a list item applies to your research, read the appropriate section before selecting a response.

Materials & experimental systems

n/a	Involved in the study	
☐	☒	Antibodies
☐	☒	Eukaryotic cell lines
☒	☐	Palaeontology and archaeology
☒	☐	Animals and other organisms
☒	☐	Clinical data
☒	☐	Dual use research of concern
☒	☐	Plants

Methods

n/a	Involved in the study	
☒	☐	ChIP-seq
☒	☐	Flow cytometry
☒	☐	MRI-based neuroimaging

Antibodies

Antibodies used	Antibodies used for immunoblotting:

Antibodies used

anti-β-actin (Sigma, A5316), anti-CSK (Cell Signaling Technology, #4980), anti-KCTD5 (Proteintech, 15553-1-AP), anti-NF1 (abcam, ab17963), anti-NF2 (Cell Signaling Technology, #6995), anti-PTEN (Cell Signaling Technology, #9559), anti-YAP1 (Cell Signaling Technology, #14074), anti-pYAP1 S127 (Cell Signaling Technology, #13008), anti-WWTR1 (Cell Signaling Technology, #70148), anti-p-EGFRY1068 (Cell Signaling Technology, #2234), anti-EGFR (Cell Signaling Technology, #4267), anti- p-AKTS473 (Cell Signaling Technology, #4060), anti-AKT (Cell Signaling Technology, #9272), anti-p-MEK1/2S217/221 (Cell Signaling Technology, #9154), anti-MEK1/2 (Cell Signaling Technology, #4694), anti-p-ERK1/2T202/Y204 (Cell Signaling Technology, #9106), anti-ERK1/2 (Cell Signaling Technology, #9102), anti-MET (Cell Signaling Technology, #8198), anti-GAPDH (Cell Signaling Technology, #2933), anti-mouse-IgG-HRP (Cell Signaling Technology, #7076) and anti-rabbit-IgG-HRP (Cell Signaling Technology, #7074)
Antibodies used for immunofluorescence:
(phospo-p44/42 (Cell Signaling Technology, mAb E10 #9106), phospho-AKT (Cell Signaling Technology, RmAb, D9E #4075, AF647 conjugated), phospho-S6 (Cell Signaling Technology, RmAb, D57.2 #9865, AF594 conjugated)), anti-YAP1/WWTR1 (Cell Signaling Technology, mAb D24E4, #8418) and AlexaFluor 488 anti-mouse or AlexaFluor 647 anti-rabbit (both Thermo-Fisher, Waltham, MA).
Antibodies used for immunohistochemistry:
Anti-YAP1 D8H1X antibody (#14074 CST, MA, USA) and anti-WWTR1 E8E9G antibody (#83669, CST, MA, USA)

Validation

All antibodies are commercially available. Manufacturers website provide relevant information regarding antibody validation.

Eukaryotic cell lines

Policy information about cell lines and Sex and Gender in Research

Cell line source(s)

PC-9, HCC4006 cell lines were purchased from ATCC and HCC827 cell line from ECACC.

Authentication

Authentication was done by STR profilling.

Mycoplasma contamination

Cell lines did undergo regular Mycoplasma testing.

Commonly misidentified lines
(See ICLAC register)

Name any commonly misidentified cell lines used in the study and provide a rationale for their use.
